# Supplementary material for: Magnetic bead-sensitized optoporation coupled with antibodies-based activation for mRNA CAR-T cell manufacturing
Source: Mol Ther Methods Clin Dev. 2025 Feb 4;33(1):101428. doi: 10.1016/j.omtm.2025.101428 (PMC11910140; doi:10.1016/j.omtm.2025.101428)
Supplement: Document S1. Figures S1–S9 and Table S1 [file mmc1.pdf]

OMTM, Volume *33*

Supplemental information

Magnetic bead-sensitized optoporation coupled with antibodies-based activation for mRNA CAR-T cell manufacturing

Noelia Maldonado-Pérez, Marie-Agnès Doucey, Dzhangar Dzhumashev, Darel Martínez Bedoya, Luis Castillo Cantero, Caroline Boudousquie, Yann Pierson, Luc Henry, Valérie Dutoit, and Denis Migliorini

Table S1. Summary of the optoporation parameters used after optimization.

Delivery parameter	**Range**	**Optimal condition**
Pulse energy	1 - 90 µJ	90 µJ
Repetition rate	100 - 1000 Hz	1000 Hz
Scanning speed	10 - 200 mm/s	80 mm/s
Longitudinal spot location	0 - 18 mm	13.9 mm
Diameter lens	1.2 - 9.6 mm	1.2 mm
Spot size	1.32 - 10.58 µm	10.58 µm

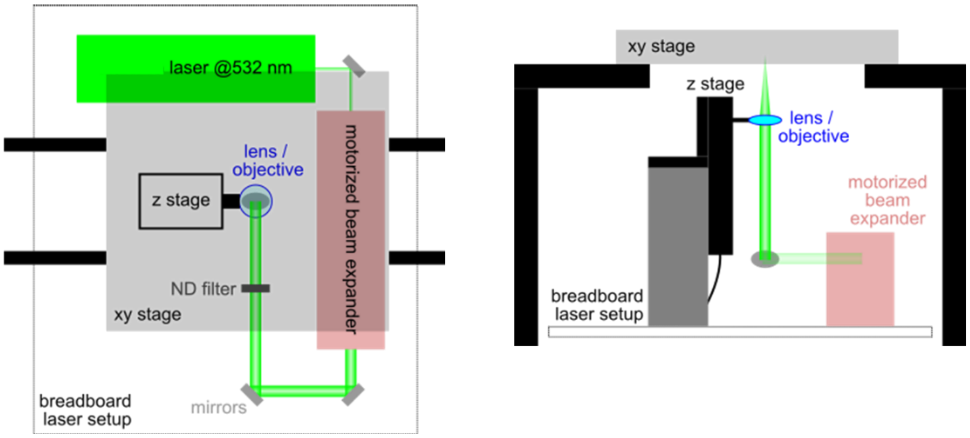

Figure S1. Optical scheme of the optoporation platform. The xy stage and laser setup are on separate breadboards. The laser setup counts with a beam expander, a lens, a ND filter to allow very low pulse energies (nJ range or below) and three mirrors.

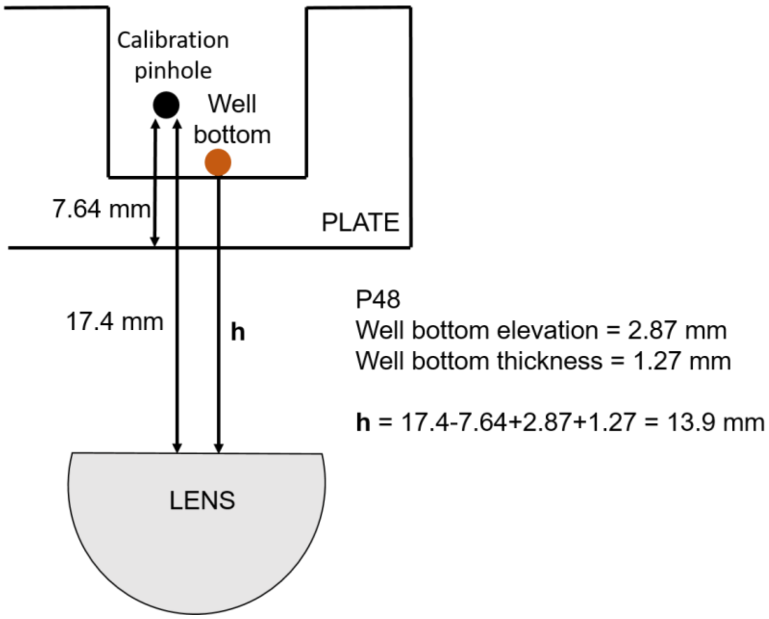

Figure S2. **Longitudinal spot location (LSL) estimation to locate the laser spot at the bottom of the well of a 48 well plate.** The input value for LSL is the result of the calculation of distance from lens to well bottom (h), which is based on the reference distances to the pinhole provided by the manufacturer.

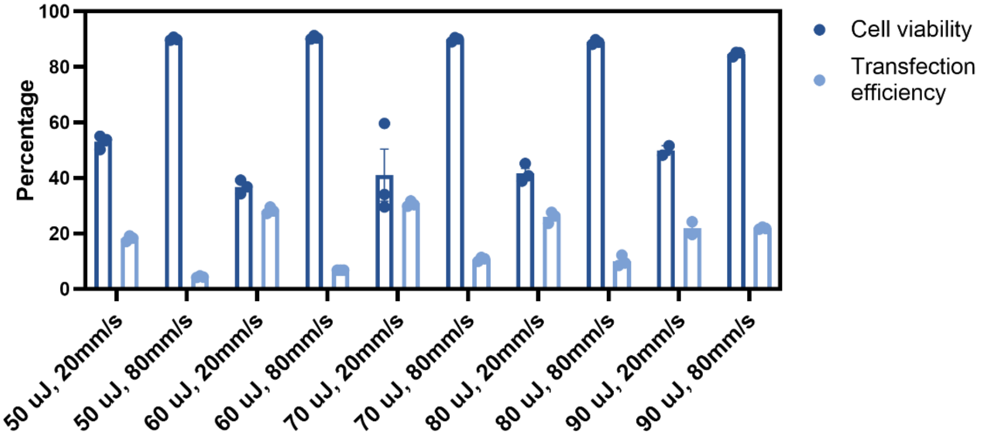

Figure S3. Optimization of scanning speed and energy pulse. Cell viability (dark blue) measured by flow cytometry 24 hours post-optoporation by staining of cells with a viability marker and efficiency of optoporation (light blue) measured by flow cytometry and reflected by the percentage of eGFP+ cells. Shown are mean cell viability and optoporation efficiency from 3 technical replicates ± SEM.

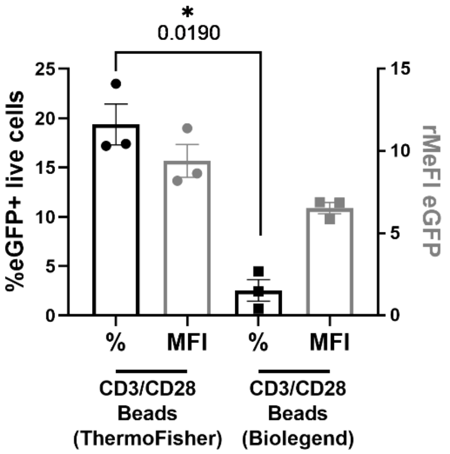

Figure S4. Magnetic bead-sensitized optoporation efficiency is dependent on the origin of CD3/CD28 magnetic beads. Primary human T cells were activated with human CD3/CD28 Dynabeads from ThermoFisher or human CD3/CD28 T Cell Activation Beads from Biolegend before optoporation of eGFP-mRNA. Transfection efficiency 24 hours post-optoporation is expressed as the percentage of eGFP+ live cells (left y-axis, black) and the relative median fluorescence intensity (rMeFI) relative to eGFP- cells (right y-axis, grey), n=3 independent donors. Data are shown as mean ± SEM.

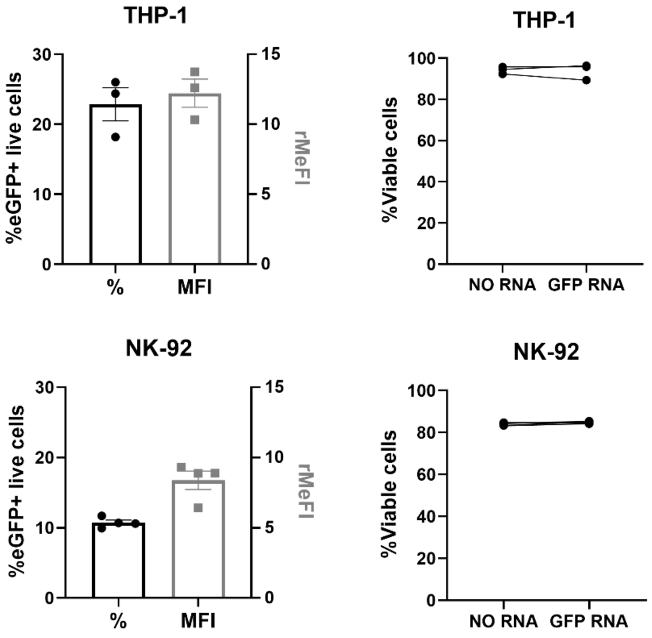

Figure S5. Monocyte and NK cell lineages are efficiently optoporated. THP-1 cells (top row) and NK cells (bottom row) optoporation efficiency and viability were measured by flow cytometry. Transfection efficiency expressed as percentage of eGFP+ cells among live cells (left y-axis, black) and relative median fluorescence intensity (rMeFI) relative to eGFP- cells (right y-axis, grey) is shown 24 hours post-optoporation. Cell viability was measured 24 hours post-optoporation by staining with a cell viability marker Data are shown as mean ± SEM.

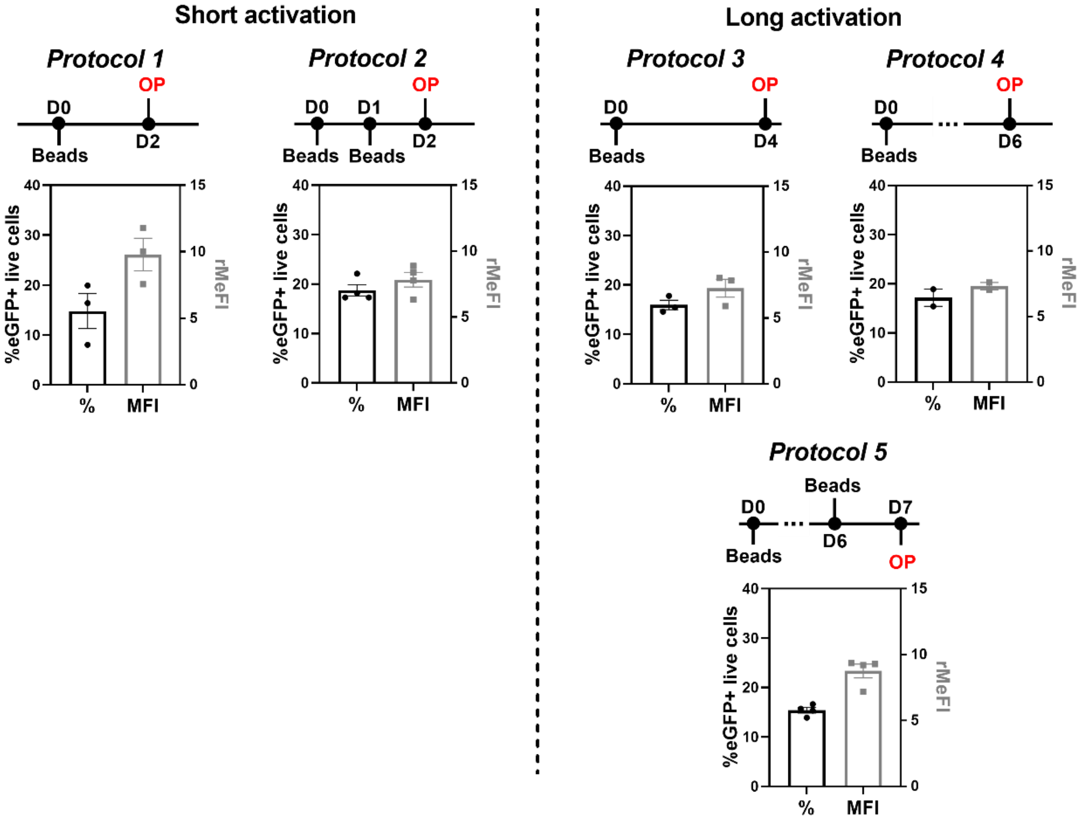

Figure S6. Impact of T cell activation time on mRNA optoporation efficacy. Primary human T cells were activated with CD3/CD28 Dynabeads (Beads) for different times before performing optoporation (OP) of eGFP-mRNA. Transfection efficiency 24 hours post-optoporation is expressed as the percentage of eGFP+ live cells (left y-axis, black) and the relative median fluorescence intensity (rMeFI) relative to eGFP- cells (right y-axis, grey) of different conditions. Data are shown as mean ± SEM.

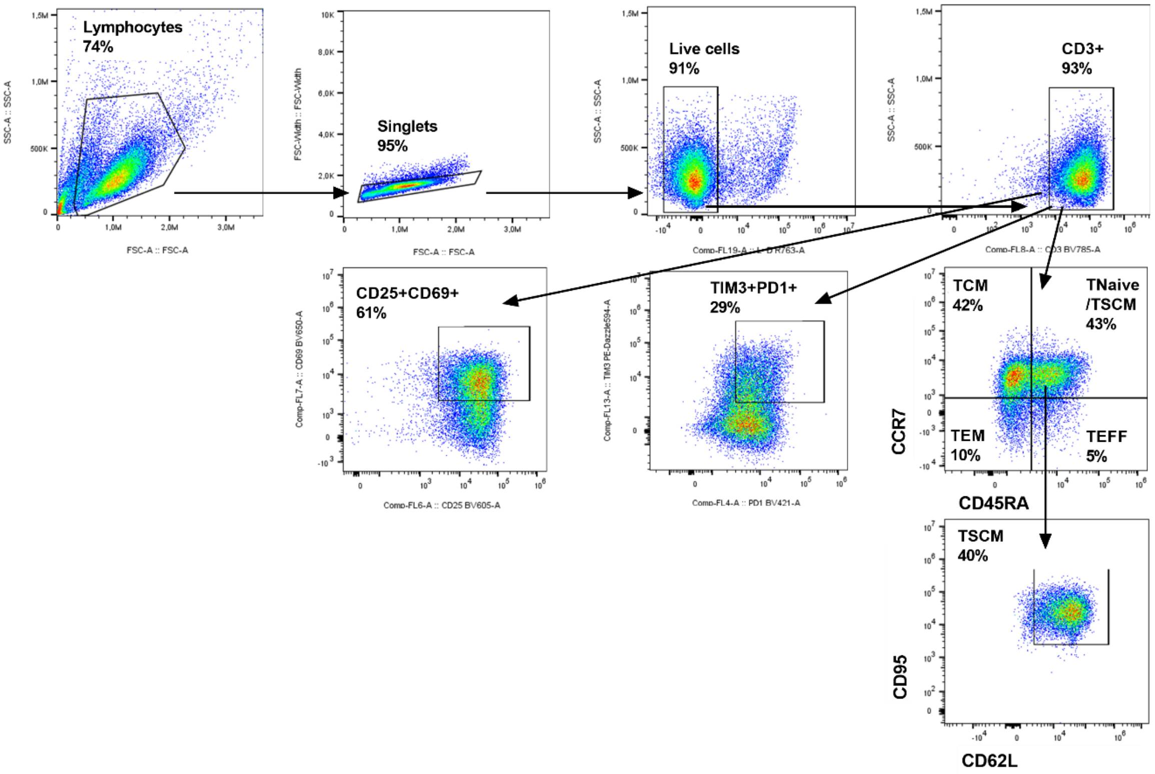

Figure S7. Gating strategy. Flow cytometry data analysis followed to obtain the different T cell populations.

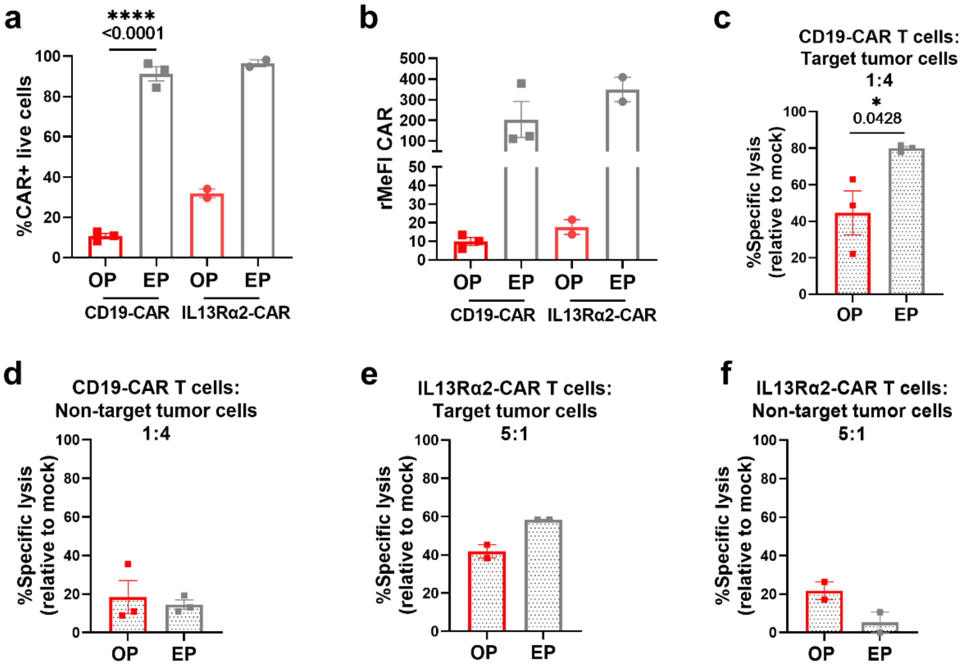

Figure S8. **Generation of different functional CAR T cells through mRNA optoporation. a)** Percentage of CAR+ T cells (left) and **b)** relative median fluorescence intensity (rMeFI) relative to CAR- cells (right) after optoporation (OP) and electroporation (EP) with αCD19-CAR-mRNA and αIL13Rα2-CAR-mRNA, n=2-3 independent donors. **c)** Percentage of specific lysis of αCD19-CAR-T cells generated by optoporation (OP) or electroporation (EP) (diluted to obtain the same percentage of CAR+ T cells) against target tumor cells (Nalm6) and **d)** non-target tumor cells (Jurkat) at E:T ratio 1:4 determined by an IncuCyte S3 system at 40 h of co-culture, n=3 independent donors. **e)** Percentage of specific lysis of αIL13Rα2-CAR-T cells generated by optoporation (OP) or electroporation (EP) (diluted to obtain the same percentage of CAR+ T cells) against target tumor cells (Ge518-WT) and **f)** non-target tumor cells (GE738) at E:T ratio 5:1 determined by an IncuCyte S3 system at 40 h of co-culture, n=2 independent donors. Data are shown as mean ± SEM. Statistics are based on paired, two-tailed Student´s t-test (a,c), *p<0.05, ****p<0.0001. The percentage of specific lysis was calculated according to the formula: (0-100)/(GFP area at 40h/0h in presence of mock T cells-0)*(GFP area at 40h/0h in presence of CAR-T cells)+100.

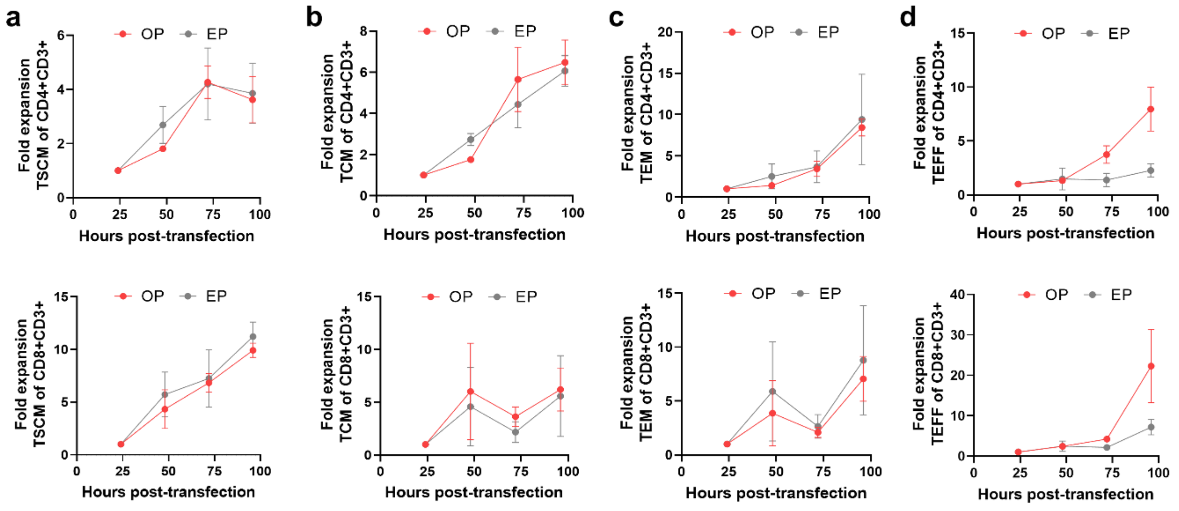

Figure S9. **Frequencies of T cell subpopulations in CAR-T cells generated via optoporation are comparable to electroporation. a)** Fold expansion of stem cell memory T cells (TSCM) CD4+CD3+ (upper graph) and TSCM CD8+CD3+ (lower graph) over time after optoporation (OP) or electroporation (EP) of αPTPRZ1-CAR mRNA. **b)** Fold expansion of central memory T cells (TCM) CD4+CD3+ (upper graph) and TCM CD8+CD3+ (lower graph) over time after optoporation (OP) or electroporation (EP) of αPTPRZ1-CAR mRNA. **c)** Fold expansion of effector memory T cells (TEM) CD4+CD3+ (upper graph) and TEM CD8+CD3+ (lower graph) over time after optoporation (OP) or electroporation (EP) of αPTPRZ1-CAR mRNA. **d)** Fold expansion of effector T cells (TEFF) CD4+CD3+ (upper graph) and TEFF CD8+CD3+ (lower graph) over time after optoporation (OP) or electroporation (EP) of αPTPRZ1-CAR mRNA. Cell counts were determined by Trypan blue exclusion and normalized to the initial number of seeded cells at 24 hours post-transfection, n=3 independent donors. Data are shown as mean ± SEM.
