# Supplementary material for: Magnetic bead-sensitized optoporation coupled with antibodies-based activation for mRNA CAR-T cell manufacturing
Source: Mol Ther Methods Clin Dev. 2025 Feb 4;33(1):101428. doi: 10.1016/j.omtm.2025.101428 (PMC11910140; doi:10.1016/j.omtm.2025.101428)
Supplement: Document S2. Article plus supplemental information [file mmc2.pdf]

Molecular Therapy
Methods & Clinical Development
Original Article

Magnetic bead-sensitized optoporation coupled with antibodies-based activation for mRNA CAR-T cell manufacturing

Noelia Maldonado-Pérez,[1,2] Marie-Agnès Doucey,[5] Dzhangar Dzhumashev,[1,2] Darel Martínez Bedoya,[1,2] Luis Castillo Cantero,[1,2] Caroline Boudousquie,[5] Yann Pierson,[5] Luc Henry,[5] Valérie Dutoit,[1,2] and Denis Migliorini[1,2,3,4]

[1]Center for Translational Research in Onco-Hematology, University of Geneva, 1211 Geneva, Switzerland; [2]Department of Oncology, University Hospital of Geneva, 1205 Geneva, Switzerland; [3]Swiss Cancer Center Léman, Lausanne, Switzerland; [4]Agora Cancer Research Center, 1005 Lausanne, Switzerland; [5]Limula SA, 1066 Épalinges, Switzerland

Immunotherapy is facing a revolution with the advent of immune cell engineering. Chimeric antigen receptor (CAR)-T cell therapy has shown unprecedented efficacy in B cell malignancies and is now being evaluated in other disease areas. Viral transduction is the most common method for immune cell genetic engineering, but presents important limitations, such as high reagent costs and regulatory concerns due to mutagenesis risk. One prevailing non-viral gene delivery strategy relies on the electroporation of non-integrating RNA. However, most modern electroporation technologies also require high reagent costs and rely on the use of proprietary software and transfection buffers. Nanoparticle-sensitized optoporation represents an alternative method for transient permeabilization of cells. Here, we introduce magnetic bead-sensitized optoporation, in which commercially available superparamagnetic beads coupled with anti-human CD3 and CD28 antibodies are used as photosensitizers for efficient genetic cargo delivery into human primary T cells and other immune cells. We show that magnetic bead-sensitized optoporation of human T cells generates functional mRNA-based CAR-T cells without affecting T cell product memory phenotype or activation potential. Importantly, optoporated T cells exhibited a greater proliferation capacity relative to electroporated T cells. In conclusion, our findings suggest that magnetic bead-sensitized optoporation holds promise as mRNA delivery strategy for immune cell therapy.

INTRODUCTION

Engineered T cells generated using gene transfer technologies to specifically target tumor antigens have revolutionized the field of adoptive cell therapy. One prominent example is chimeric antigen receptor (CAR)-T cell therapy, a personalized treatment that has demonstrated remarkable efficacy in B-cell acute lymphocytic leukemia, non-Hodgkin lymphoma, and multiple myeloma and led to the approval of six CAR-T cell products by the European and American regulatory authorities.[1] These treatments currently rely on stable genetic modifications of patients' cells using γ-retroviruses or lentiviruses that randomly integrate an exogenous sequence into the cell genome. This approach has several limitations. First, the complex and low-yielding γ-retroviruses or lentiviruses manufacturing processes result in high reagent costs. In addition, the limited cargo capacity of viral vectors restricts the capacity to engineer CAR-T cells with multiple genes.[2,3] Although more than 30,000 patients worldwide have been treated with this approach so far, with an acceptable toxicity profile,[4,5] there has been concern that genomic integration could lead to a risk of genotoxic events. In November 2023, the U.S. Food and Drug Administration announced that several patients treated with commercially available CAR-T cell therapies were diagnosed with secondary cancers, mandating long-term safety monitoring.[6]

RNA-based transfection of CAR molecules overcomes some of these issues. RNA is non-integrative and leads to transient transgene expression at the cell surface,[7] reducing the risk of on-target, off-tumor toxicities[8] and the possibility of cell transformation. In addition, it holds the promise of fast and cost-effective manufacturing.[9–11] RNA-based transfection generally relies on electroporation, which uses electric pulses to permeabilize membranes for intracellular delivery of payloads.[12,13] However, it comes with drawbacks, such as inconsistent membrane disruption, endosomal escape, altered gene expression, and heightened cytotoxicity,[14–16] prompting the exploration of alternative transfection methods.

Optical membrane disruption, or optoporation, has emerged as an efficient method to transiently permeabilize the cell membrane using high-intensity light, allowing the delivery of payloads such as mRNA.[15–18] In pioneering studies, a pulsed laser was directly focused

Received 22 August 2024; accepted 31 January 2025;
https://doi.org/10.1016/j.omtm.2025.101428.

Correspondence: Denis Migliorini, Center for Translational Research in Onco-Hematology, University of Geneva, 1211 Geneva, Switzerland.
E-mail: denis.migliorini@unige.ch

© 2025 The Author(s). Published by Elsevier Inc. on behalf of The American Society of Gene and Cell Therapy.
This is an open access article under the CC BY license (http://creativecommons.org/licenses/by/4.0/).

on a small spot on the cell membrane to create a pore (laser-induced stress wave).[19–21] While cells could be successfully transfected, the main limitation was a very low throughput, given the fundamentally single-cell nature of the technology.[22–24] In the last decades, photothermal nanomaterial-mediated optoporation emerged, in which pulsed laser irradiation is applied to cells attached to a photothermal nanomaterial (photosensitizer), substantially increasing the throughput.[25] These photosensitizers efficiently absorb laser light and convert this energy into distinct phenomena such as local heating, photochemical reactions, acoustic shockwaves, or the formation of water vapor nanobubbles (VNBs), depending on the laser energy. Irradiation with low-intensity laser pulses results in photothermal effects, inducing pore formation by denaturation of integral membrane proteins or local phase transitions of the lipid bilayer.[26] When a nanosecond pulsed laser is used, the temperature of the photosensitizers reaches hundreds of degrees, triggering VNB formation and their subsequent collapse. This leads to high-pressure shockwaves and fluid shear stress that generate transient pores in the cell membrane.[27–31]

Gold nanoparticles (AuNPs) have often been used as photothermal sensitizers for optoporation, allowing the efficient delivery of dextran (a model macromolecule), small interfering RNA, and mRNA.[15,17,18,25,32,33] However, the impact of AuNPs on different immune cell types remains inadequately studied, raising regulatory concerns and requiring comprehensive biocompatibility testing.[34,35]

Herein, we propose a straightforward alternative to previously reported photothermal sensitizers. We designed and generated our custom-built optoporation device and provided evidence that commercially available magnetic beads can act as photosensitizers, specifically 4.5 μm superparamagnetic beads coupled with anti-human CD3 and CD28 antibodies. The CTS (Cell Therapy Systems) Dynabeads CD3/CD28 are already in use for *ex vivo* isolation, activation, and expansion of human T cells in the context of T cell manufacturing for clinical studies. To demonstrate that Dynabeads could act both as a T cell activator and a photoporation sensitizer, we tested the intracellular delivery of EGFP-mRNA and CAR-mRNA in human primary T cells. A comparative analysis of mRNA transfection was conducted using magnetic bead-mediated optoporation and electroporation using the MaxCyte GTx technology. MaxCyte electroporation is clinically validated for efficient intracellular delivery while maintaining high cell viability and is used in the production of the first non-viral cell therapy approved.[36]

RESULTS

Successful optoporation of primary human T cells with mRNA using magnetic beads

We investigated superparamagnetic beads coupled with anti-human CD3 and CD28 antibodies (CD3/CD28 Dynabeads) as sensitizers for the delivery of mRNA into cells through optoporation. CD3/CD28 bead-activated T cells were seeded in a 48-well plate and EGFP-mRNA was added (Figure 1A) before the plate was placed in the XY stage of a custom-built optoporation platform (Figures 1B and S1). Several parameters were explored to reach optimal cargo delivery (Table S1 and Figure S2). First, we studied the transfection efficiency and viability of primary human T cells using two optoporation scanning speeds (20 and 80 mm/s) and five distinct energy pulses (50, 60, 70, 80, and 90 μJ). As shown in Figures 1C and S3, cell viability was preserved at high speed, while the percentage of transfected cells was low. In contrast, a low speed resulted in a higher RNA transfection efficiency, but at the expense of cell viability. The combination of a 90 μJ energy pulse and 80 mm/s scanning speed was chosen as it offered the best trade-off between transfection efficiency (20%) and viability (>80%) and was used in all subsequent experiments, together with the optoporation parameters listed in Table S1. We then showed that optoporation in the absence of beads did not result in efficient cell transfection, likely due to the absence of membrane permeabilization. This was suggested by the lack of cell transfection in cells activated with magnetic beads and dissociated from them prior to optoporation in the presence of EGFP-mRNA (NO BEADS condition) (Figure 2). In addition, we tested slightly bigger anti-CD3/CD28 magnetic beads obtained from another vendor and observed a significant reduction in transfection efficiency compared with Dynabeads (Figure S4). To document the versatility of the method, we subjected a human acute monocytic leukemia cell line (THP-1) and natural killer lymphoma cell line (NK-92) to the same optoporation protocol and observed efficient transfection without impact on viability (Figure S5). In conclusion, we defined optimized optoporation conditions that result in the efficient transfection of multiple immune cell subsets and preserve cell viability.

T cells are efficiently optoporated after long and short activation procedures

Next, we explored whether the duration of T cell activation impacted optoporation outcome. T cells were activated with CD3/CD28 beads for short (2 days) or long (>4 days) periods, following different protocols (Figure S6). In short activation procedures, T cells were stimulated once with CD3/CD28 beads on day 0 (protocol 1) or received consecutive stimulation on days 0 and 1 (protocol 2) before performing optoporation at day 2 (Figure S6, left). In long activation procedures, we compared cells optoporated following 4 (protocol 3), 6 days (protocol 4), and 7 days (protocol 5) of activation, in the case of 7 days cells were re-stimulated at day 6 (Figure S6, right). In all procedures, EGFP-mRNA optoporation efficiency was evaluated using flow cytometry to measure two parameters: the percentage of EGFP-positive cells and the intensity of EGFP expression (reflected by the relative median fluorescence intensity of EGFP).

Protocols 2 (short activation) and 5 (long activation) provided the highest EGFP expression levels while leading to a reproducible percentage of $EGFP^+$ cells and were further compared side by side (Figure 3A). Both protocols generated a comparable percentage of EGFP-positive cells, reaching a mean of 18.2% (protocol 2) and 15.3% (protocol 5). Of note, EGFP expression was transient and the percentage of $EGFP^+$ cells dropped to 7.5% and 4% at 3 days after optoporation, respectively (Figure 3B). Cell viability was slightly impacted using the long activation procedure but it was restored after 3 days (Figure 3C). Regarding the composition of T cell subsets, a higher percentage of T stem cell

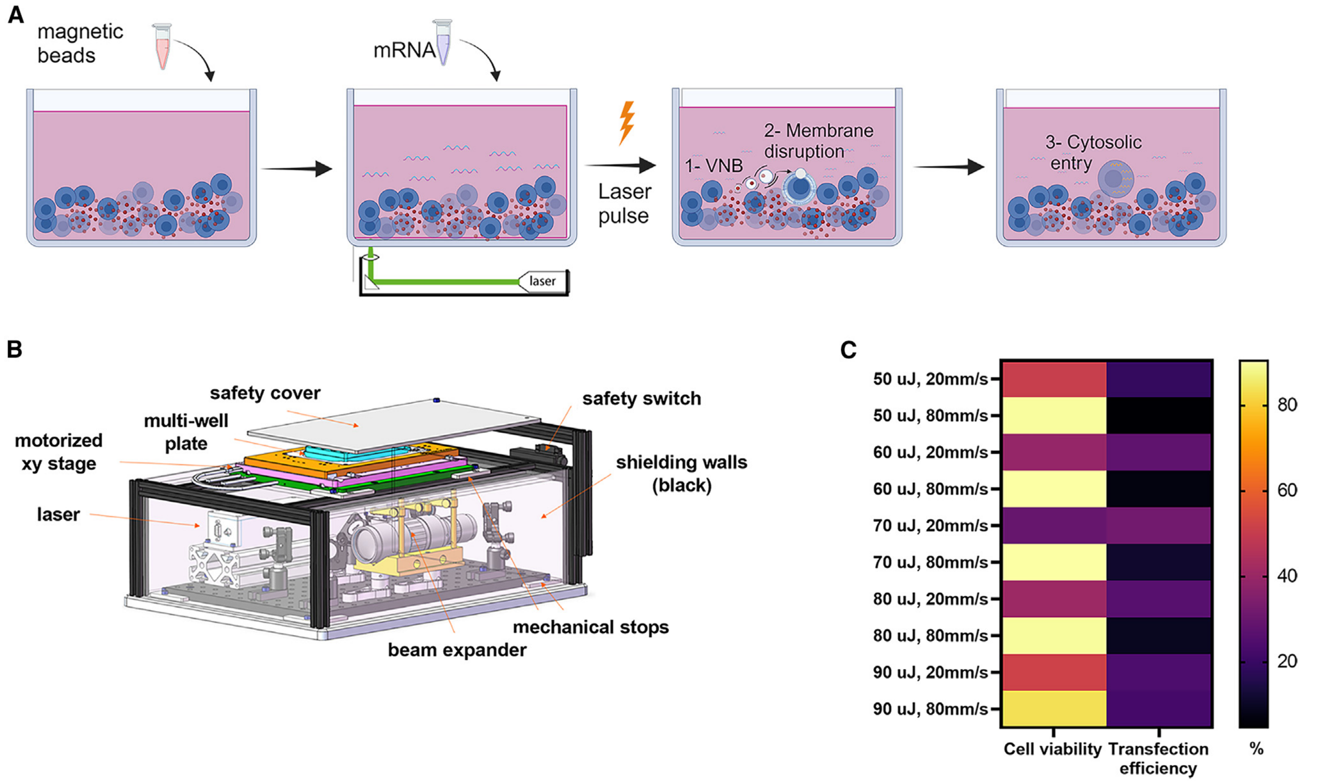

Figure 1. Optoporation principle and optimization

(A) Schematic representation of magnetic bead-mediated optoporation for mRNA delivery into cells. mRNA molecules are added to T cells (blue) in the presence of magnetic beads (red) and the mix is irradiated with a nanosecond pulsed-laser. Transient permeabilization of the cells is induced by collapsing of the nanobubbles allowing mRNA molecules to enter the cells. (B) Design of the custom-built optoporation device used for laser-based transfection in multi-well plates. (C) Heatmap representing viability and transfection efficiency (% EGFP$^+$ cells) determined 24 h after optoporation by flow cytometry. Mean optoporation efficiency and cell viability from three technical replicates are shown.

memory (TSCM) cells was obtained using the short activation protocol while a higher percentage of T effector memory (TEM) cells was obtained with the long activation procedure (Figure 3D). Interestingly, this pattern of T cell differentiation was observed in both transfected (EGFP$^+$) (Figure 3E) and non-transfected (EGFP$^-$) (Figure 3F) T cells, and thus more likely to be associated with the activation rather than with the optoporation procedure. Given the fact that T cell products containing less differentiated CAR-T cells have been correlated with a superior anti-tumor response in several preclinical and clinical studies,[37–40] we selected the short activation protocol for further investigations. Finally, with the aim of improving transfection efficiency, we tested whether incubating the T cells in the plate and allowing them to sediment for 20 min could positively affect optoporation performance. Cell sedimentation tremendously impacted transfection efficiency and doubled the frequency of EGFP$^+$ cells (Figure 3G). Cell viability was impacted during the first 24 h after optoporation (Figure 3H), but was restored at 72 h (Figure 3I).

Optoporation does not alter T cell phenotype and function

In addition to obtaining efficient cargo delivery, withholding of T cell activation potential is an important determinant for selecting a transfection method.[41] Therefore, we investigated the T cell activation status after magnetic bead-sensitized optoporation and compared it with that of electroporated T cells. To unambiguously investigate the effects of both processes, stimulated T cells were optoporated or electroporated in the absence of cargo and rested overnight. The following day, cell viability, cell growth, memory, activation, and exhaustion phenotypes were assessed. First, we evaluated cell viability and cell proliferation, which contribute to an effective immune response.[39,41] Although the optoporation process initially affected cell viability within the first 24 h as compared with electroporated cells, it was restored after 4 days of culture (Figure 4A). Notably, optoporated T cells displayed an improved growth rate at day 4 compared with electroporated T cells (Figure 4B).

As an undifferentiated T cell phenotype correlates with prolonged therapeutic responses,[37–40] we looked at the frequency of less differentiated TSCM cells (CD45RA$^+$, CCR7$^+$, CD62L$^+$, and CD95$^+$) (see gating strategy, Figure S7). As shown in Figure 4C, optoporated T cells presented a slightly superior frequency of TSCM compared with non-treated or electroporated T cells 24 h after transfection. Given the high variability observed, we cannot conclude that

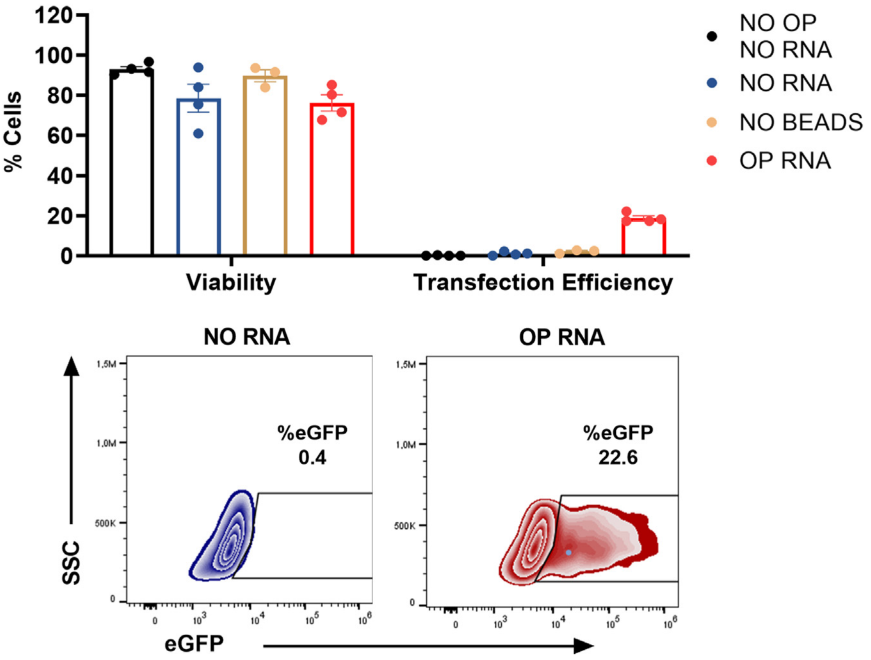

Figure 2. EGFP is expressed in primary human T cells following optoporation in the presence of magnetic beads

(Top) Cell viability measured 24 h after optoporation by staining of cells with a viability marker (left) and efficiency of optoporation measured by flow cytometry and reflected by the percentage of $EGFP^+$ cells (right), $n = 4$ independent donors. NO OP, NO RNA, untreated control (no optoporation, no mRNA); NO RNA, mock optoporation control (laser pulse with magnetic beads only); NO BEADS, mock mRNA control (laser pulse in the presence of mRNA and in the absence of magnetic beads); OP RNA, laser pulse in the presence of mRNA and magnetic beads. (Bottom) Representative flow cytometry density plots after optoporation of human T cells without (NO RNA) and with (OP RNA) mRNA encoding for EGFP.

optoporation modifies significantly the T cell memory phenotype. After 4 days, lower and comparable levels of TSCM were observed in all conditions. To evaluate the exhaustion and activation state of T cells, we assessed the expression of the immune checkpoints PD1 and TIM3, as well as the activation markers CD25, CD69, and CD95 (see gating strategy, Figure S7). The percentages of $CD25^+$, $PD1^+$, $CD95^+$, $CD25^+CD69^+$, and $PD1^+TIM3^+$ T cells were comparable in all experimental conditions; with very low levels being observed after 4 days of culture, except for CD95 (Figures 4D and 4E). Finally, no significant differences were observed in the production of tumor necrosis factor (TNF)-α, interferon (IFN)-γ, and interleukin (IL)-2 cytokines or of granzyme B by expanded and re-stimulated optoporated T cells when compared with electroporated or non-treated T cells (Figure 4F). Altogether, these results suggest that T cells that suffer magnetic bead-mediated optoporation can be functional upon reactivation *in vivo*.

Optoporation generates functional mRNA-based CAR-T cells

Based on these results, we generated mRNA-based CAR-T cells using magnetic bead-based optoporation. We used a CAR directed to the antigen PTPRZ1, previously identified by our group as a relevant tumor-associated cell surface protein in glioblastoma (GBM)[42,43] and used as part of the IMA950 multi-peptide vaccine in phase I/II clinical trials.[44] We generated CAR-T cells (approximately 25% CAR^+) using the optoporation protocol, but efficiency was significantly lower compared with CAR delivery via electroporation, both in terms of the percentage of CAR^+ cells and CAR expression levels (Figure 5A). This resulted in a faster loss of CAR expression on the cell surface, becoming almost undetectable after 3 days (Figure 5B). Furthermore, optoporation, but not electroporation, had an impact on CAR-T cell viability at the earliest time point (Figure 5C). However, optoporated T cells displayed an improved growth rate compared with electroporated T cells (Figure 5D). One day after transfection, anti-tumor activity was assessed against the Ge518 human GBM cell line engineered to express the antigen, using the same number of CAR^+ T cells in the optoporated and electroporated conditions. After 72 h of co-culture, optoporated CAR-T cells were able to trigger lysis of antigen-expressing tumor cells in a specific manner, although to a lesser extent as compared with electroporated T cells, probably due to the lower levels of CAR expression (Figure 5E). This result was consistent with the cytokine secretion profile observed after 72 h of co-culture with target cells, in which CAR-T cells generated via electroporation (CAR EP) exhibited significantly higher IL-2 release compared with optoporated CAR-T cells (CAR OP), and an overall trend of CAR EP cells to secrete higher levels of cytokines, particularly IL-6, TNF-α, granzyme B, and granzyme A (Figure 5F). Additionally, we demonstrated the feasibility of the optoporation protocol to generate functional CAR-T cells targeting other antigens used in clinical trials such as CD19 and IL13Rα2 (Figure S8).

We compared the phenotype of unstimulated CAR-T cells generated via optoporation and electroporation. No significant differences were observed in the CD8/CD4 ratio between the two methods (Figure 6A). Furthermore, optoporated and electroporated CAR-T cells showed comparable $CD8^+CD3^+$ and $CD4^+CD3^+$ memory T cell populations over time in culture, both in terms of percentage and total cell counts (Figures 6B and S9). To assess the exhaustion phenotype, we analyzed the absence of (0), expression of one (1), and co-expression of two (2) or three (3) inhibitory receptors (TIM3, PD1, LAG3). An increase in the co-expression of two exhaustion markers was observed in both $CD4^+$ and $CD8^+$ populations during the early hours after transfection for both optoporated and electroporated CAR-T cells. However, 72 h after transfection, the expression of exhaustion markers had decreased in all conditions (Figure 6C).

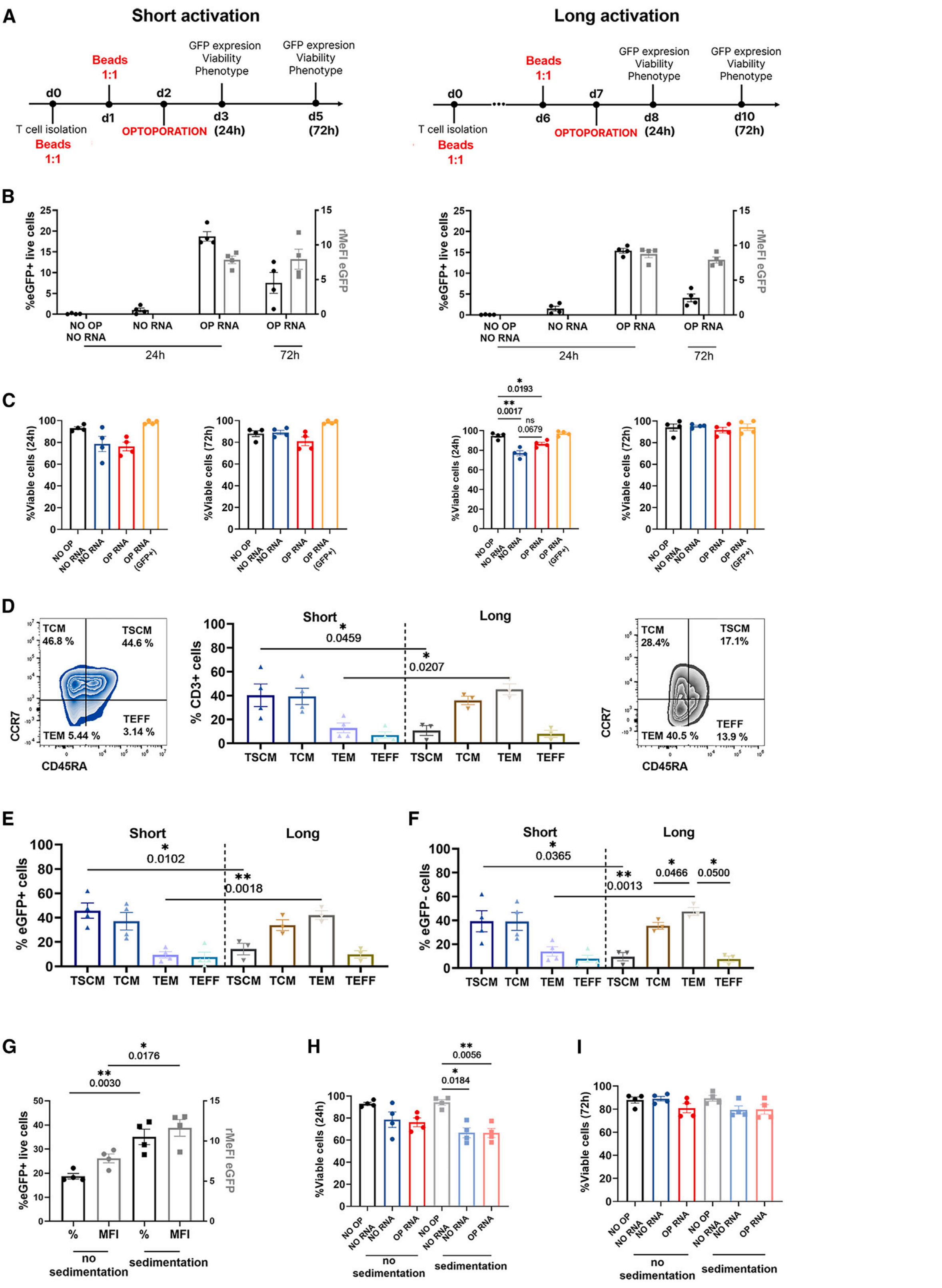

(legend on next page)

DISCUSSION

The development of new technologies in the field of gene therapy has paved the way to develop alternative opportunities for engineered cellular therapies. Viral vectors are currently the clinical and commercial standard for the generation of cell therapy products, but they face multiple issues, such as the potential for T cell transformation, high cost, and limited payload. Indeed, recently, cases of T cell lymphoma and other secondary primary malignancies after commercial CAR-T cell therapy have been reported,[6] even if recent reports were reassuring in that matter.[5] This risk may be due to several factors, such as the integration of the CAR molecule in oncogenic driving regions or the intense immune response resulting from the therapy. Long-term surveillance is crucial to gain a better understanding of this risk and minimize potential adverse consequences. In addition, viral vectors present highly variable transduction efficiencies.[41] As a consequence, mRNA-based cell therapies are actively pursued as a safer and cheaper alternative to integrative vectors.[9] Various transfection strategies have been used for mRNA delivery, from membrane disruption-mediated to carrier-mediated approaches, with electroporation being the most established non-viral transfection method.

Here, we studied the use of light-generated membrane disruption (optoporation) to deliver mRNA molecules into T cells. We report on the use of magnetic beads (Dynabeads) as sensitizers for optoporation, achieving similar mRNA transfection efficiencies as other optoporation systems described. Raes et al.[15] described a transfection efficiency of approximately 30% in Jurkat cells with 60% viability using 60 nm AuNPs as photosensitizers. However, only the study conducted by Harizaj et al.[45] showed effective delivery of mRNA in primary human T cells using polydopamine nanoparticles, with an mRNA transfection efficiency of 29% and cell viability of 63%. Using primary human T cells, we achieved a comparable frequency of EGFP$^+$ cells and a higher transgene expression level compared with Harizaj's work, without the need of adding an extra reagent to cells since Dynabeads were already present in the culture for T cell activation.

While magnetic bead-mediated optoporation achieved reasonable mRNA delivery efficiencies in T cells, there is a limitation in intracellular delivery of larger cargos as plasmid DNA. Indeed, the efficacy of optoporation is highly linked to the conditions used and remains to be optimized for the type of vectors under study and further explored for considerable sized cargos.

In addition to delivery efficiency, the long-term effects of the delivery technology on cell phenotype and function must be considered. Here, we show that transient permeabilization does not have detrimental effects on memory, activation and exhaustion markers, proliferation capacity, or secretion of the pro-inflammatory cytokines or granzyme B.

Next, we studied how this method can be exploited for intracellular delivery of CAR-mRNA. We obtained delivery efficiencies of up to 30% and cell viability from 65% to 85% in primary human T cells. In addition, we demonstrated that the generated CAR-T cell products preserved cytotoxicity capacity. To our knowledge, there are no other studies that use laser-mediated transfection to produce functional CAR-T cells. However, delivery efficiency using optoporation is not yet comparable with that obtained by electroporation. Compared with other studies suggesting a significant cytotoxic effect induced by electroporation,[14–16,46] our findings indicate that some commercially available electroporators are highly optimized for mRNA delivery, with more than 90% of cells exhibiting positive and high levels of expression, alongside cell viability exceeding 80%. Of note, we observed a diminished cell proliferation capacity in electroporated cells. A more comprehensive study would be needed to explore more precisely the short- and long-term effects of both techniques in primary cells.

In addition to its application in T cells, we tested the optoporation protocol in other clinically relevant cell types, including NK cells and THP-1 cells. Our results demonstrate that our optoporation platform effectively transfects these cell types, maintaining a high viability after transfection. This broadens the potential applicability of the method to various cell-based therapies beyond CAR-T cell treatments.

Regarding scalability, the optoporation protocol shows promise for adaptation to larger-scale manufacturing. The ability to scale the

Figure 3. T cells are efficiently optoporated following long and short activation procedures

(A) T cells were stimulated with CD3/CD28 beads for short (left, protocol 2, see Figure S6) or long (right, protocol 5, see Figure S6) periods before optoporation with EGFP-mRNA. EGFP expression, cell viability, and T cell phenotype were determined by flow cytometry. (B) Transfection efficiency expressed as percentage of EGFP$^+$ cells among live cells (left y axis, black) and relative median fluorescence intensity (rMeFI) relative to EGFP$^-$ cells (right y axis, gray) are shown 24 h and 72 h (only for OP RNA condition) after optoporation, $n = 4$ independent donors. (C) Cell viability measured 24 h and 72 h after optoporation by staining with a cell viability marker, $n = 4$ independent donors. (D) Distribution of T cell subsets in total (CD3$^+$) T cells 24 h after optoporation with EGFP-mRNA (OP RNA condition), $n = 4$ and $n = 3$ independent donors in short and long activation procedures, respectively. Representative dot plots showing the percentage of TSCM, TCM, TEM, and TEFF cells in CD3$^+$ T cells following short (left) or long (right) activation protocols. (E) Distribution of T cell subsets in EGFP$^+$ and (F) EGFP$^-$ T cells 24 h after optoporation with EGFP-mRNA, $n = 4$ and $n = 3$ independent donors in short and long activation procedures, respectively. (G) Transfection efficiency 24 h after optoporation expressed as the percentage of EGFP$^+$ live cells (left y axis, black) and the relative median fluorescence intensity (rMeFI) relative to EGFP$^-$ cells (right y axis, gray) of T cells optoporated using protocol 2 (short activation) without (no sedimentation) or with cell sedimentation (sedimentation) to the plate, $n = 4$ independent donors. (H) Percentage of viable cells after 24 h and (I) 72 h after optoporation using protocol 2 (short activation) without (no sedimentation) or with cell sedimentation (sedimentation) to the plate, determined by staining with a cell viability marker, $n = 4$ independent donors. NO OP, NO RNA, no optoporation; no mRNA; NO RNA, laser pulse with magnetic beads only; OP RNA, laser pulse in the presence of mRNA and magnetic beads. TSCM, CD45RA$^+$CCR7$^+$CD62L$^+$CD95$^+$; T central memory (TCM), CD45RA$^-$CCR7$^+$; TEM, CD45RA$^-$CCR7$^-$ and T effector (TEFF), CD45RA$^+$CCR7$^-$. Data are shown as mean ± SEM. Statistics are based on one-way ANOVA, Tukey's multiple comparisons test (C, H); on unpaired, two-tailed Welch's t test (D–F), and unpaired, two-tailed Student's t test (G), *<0.05, ** $p < 0.01$, *** $p < 0.001$.

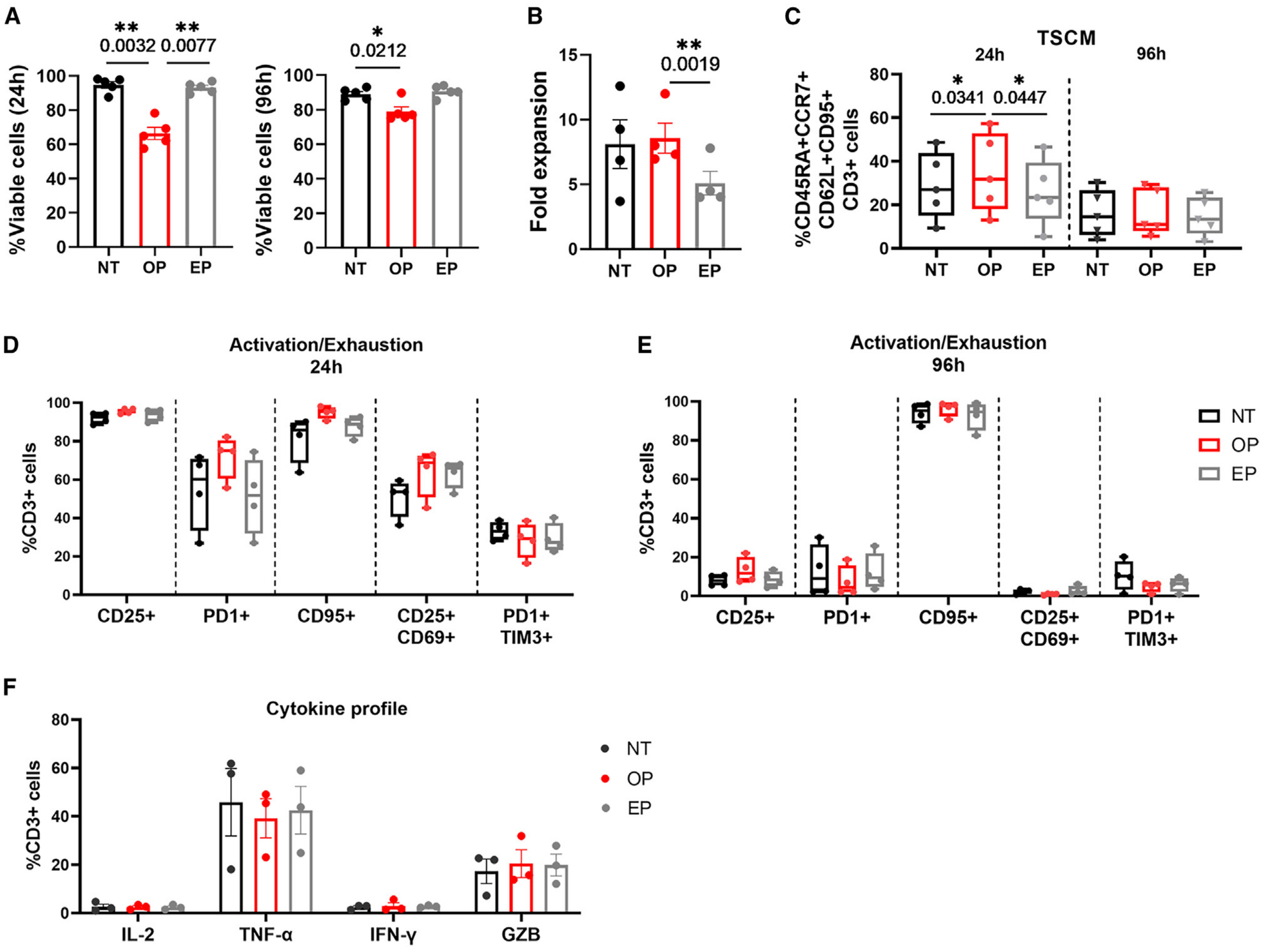

Figure 4. Optoporation does not significantly alter T cell functional phenotype

(A) Viability of T cells in the absence of treatment (NT) and following optoporation (OP) or electroporation (EP) without cargo, after 24 (left) and 96 h (right), *n* = 5 independent donors. (B) T cell fold expansion under the different conditions 4 days after transfection. Cell counts were determined by Trypan blue exclusion and normalized to the initial number of seeded cells, *n* = 4 independent donors. (C) Cumulative data showing the percentage of $CD45RA^+CCR7^+CD62L^+CD95^+$ (TSCM cells) of $CD3^+$ 24 h and 96 h after optoporation (OP) or electroporation (EP) as compared with non-treated cells (NT) cells, *n* = 5 independent donors. (D and E) Cumulative data showing the percentage of $CD25^+$, $PD1^+$, $CD95^+$, $CD25^+CD69^+$, and $PD1^+TIM3^+$ of $CD3^+$ 24 h (D) and 96 h (E) after optoporation (OP) or electroporation (EP) as compared with non-treated cells (NT) cells, *n* = 4 independent donors. (F) Production of cytokines and granzyme B (GZB) by T cells at day 7 after optoporation (OP) or electroporation (EP) as compared with NT cells, *n* = 3 independent donors. Data are shown as mean ± SEM. Statistics are based on one-way ANOVA, Tukey's multiple comparisons test (A–C) $^*p < 0.05$, $^{**}p < 0.01$.

process will depend on optimizing the optical device for high-throughput applications to ensure efficient transfection of a large number of cells while maintaining product quality. With further advances in device technology and process optimization, optoporation could be a viable solution for large-scale clinical production of genetically modified immune cells.

In summary, in this study, we successfully demonstrated the effectiveness of magnetic bead-sensitized optoporation for delivering intracellular mRNA in primary human T cells, while preserving their functionality and phenotype. Specifically, we illustrated its applicability in delivering CAR-mRNA molecules into activated T cells, resulting in the generation of mRNA-CAR T cells endowed with anti-tumor capabilities. It is important to note that this is a preliminary study, and the observed transfection efficiency in a setting of transient CAR expression may not be sufficient to treat refractory malignancies. Nevertheless, optoporation could be more readily translatable to conditions in which transient low transgene expression might be sufficient for therapeutic benefit, such as autoimmune diseases. Further optimization of the optoporation method, particularly fine-tuning the optoporation setup, such as increasing the laser spot area, and improvements in transgene stability, will be necessary to extend its applicability in oncology indications.

Finally, it is worth mentioning that, while the transient nature of CAR expression using mRNA-based approaches offers advantages such as

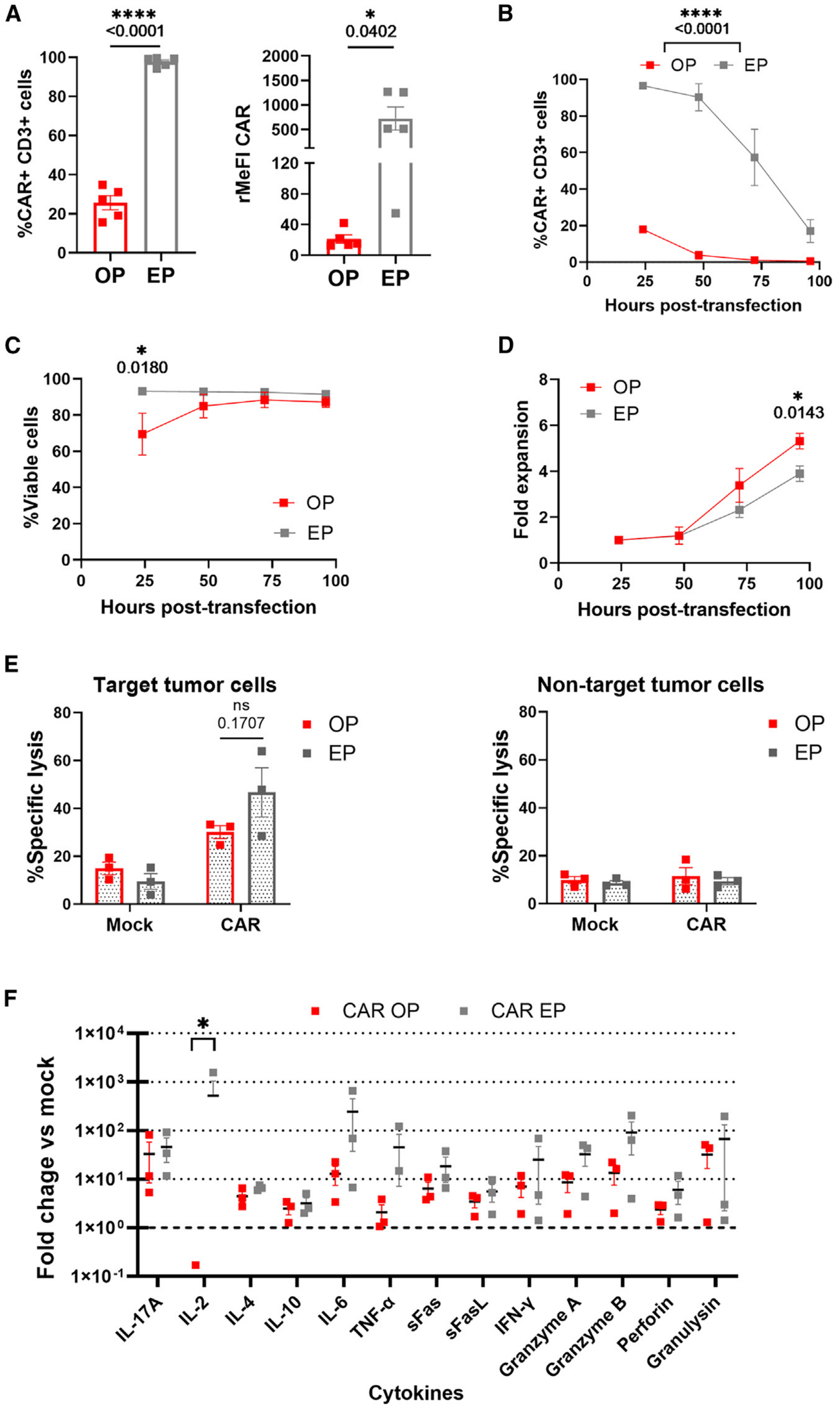

Figure 5. mRNA optoporation generates functional CAR T cells

(A) Percentage of CAR^+-T cells (left) and relative median fluorescence intensity (rMeFI) relative to CAR cells (right) after optoporation (OP) and electroporation (EP) with an αPTPRZ1-CAR-mRNA, n = 5 independent donors. (B) Percentage of CAR^+ cells over time after CAR-mRNA optoporation (OP) or electroporation (EP), n = 3 independent donors. (C) Viability of T cells transfected with CAR-mRNA over time after optoporation (OP) or electroporation (EP), n = 3 independent donors. (D) T cell fold expansion over time after CAR-mRNA optoporation (OP) or electroporation (EP). Cell counts were determined by Trypan blue exclusion and normalized to the initial number of seeded cells at 24 h, n = 3 independent donors. (E) (Left) Percentage of specific lysis of antigen-positive GBM target cells (Ge518-PTPRZ1) in co-culture with αPTPRZ1-CAR-T cells or control mock T cells generated by optoporation (OP) or electroporation (EP) (diluted to obtain the same percentage of CAR^+ T cells) as measured by flow cytometry after 72 h of co-culture. E:T ratio 1:1. (Right) Percentage of specific lysis of antigen-negative GBM target cells (Ge518-WT) in co-culture with αPTPRZ1-CAR-T cells or mock T cells generated by optoporation (OP) or electroporation (EP) (diluted to obtain the same percentage of CAR^+ T cells) as measured by flow cytometry after 72 h of co-culture. E:T ratio 1:1, n = 3 independent donors. (F) Soluble factors secreted by CAR T cells after 72 h of incubation with Ge518-PTPRZ1 cells at a 1:1 E:T ratio (fold change relative to secretion by mock EP or mock OP T cells). Data are shown as mean ± SEM. Statistics are based on paired, two-tailed Student's t test (A), and two-way ANOVA, Šídák's multiple comparisons test (B–F), $^*p < 0.05$, $^{****}p < 0.0001$.

reduced risk of insertional mutagenesis, it requires repetitive dosing to maintain therapeutic efficacy. This strategy has been associated with potential risks, including anaphylaxis due to single-chain variable fragment (scFv) immunogenicity.[47] However, immunogenicity of heavy-chain variable domain (VHH) is recognized to be reduced.[48,49] To mitigate these risks, future clinical applications should focus on optimizing dosing regimens and developing strategies to reduce immunogenicity such as scFv or VHH humanization. Moving forward, it will be valuable to explore the potential of optoporation for delivering other effector molecules, such as CRISPR-Cas9 nucleoprotein complexes for T cell engineering purposes.

MATERIALS AND METHODS

Cell culture

The GBM cell line Ge518 (wildtype [WT] or engineered to express the PTPRZ1 protein, was generated previously in the laboratory of Prof. Denis Migliorini from a patient sample) was cultured in high glucose DMEM with GlutaMAX supplemented with 10% heat inactivated fetal bovine serum (iFBS, Gibco), 5% penicillin/streptomycin (p/s, Gibco), 1 mM sodium pyruvate (Gibco), and 10 mM HEPES (Gibco). NK-92 cells (ATCC CRL-2407) were cultured in RPMI-1640 (Gibco)

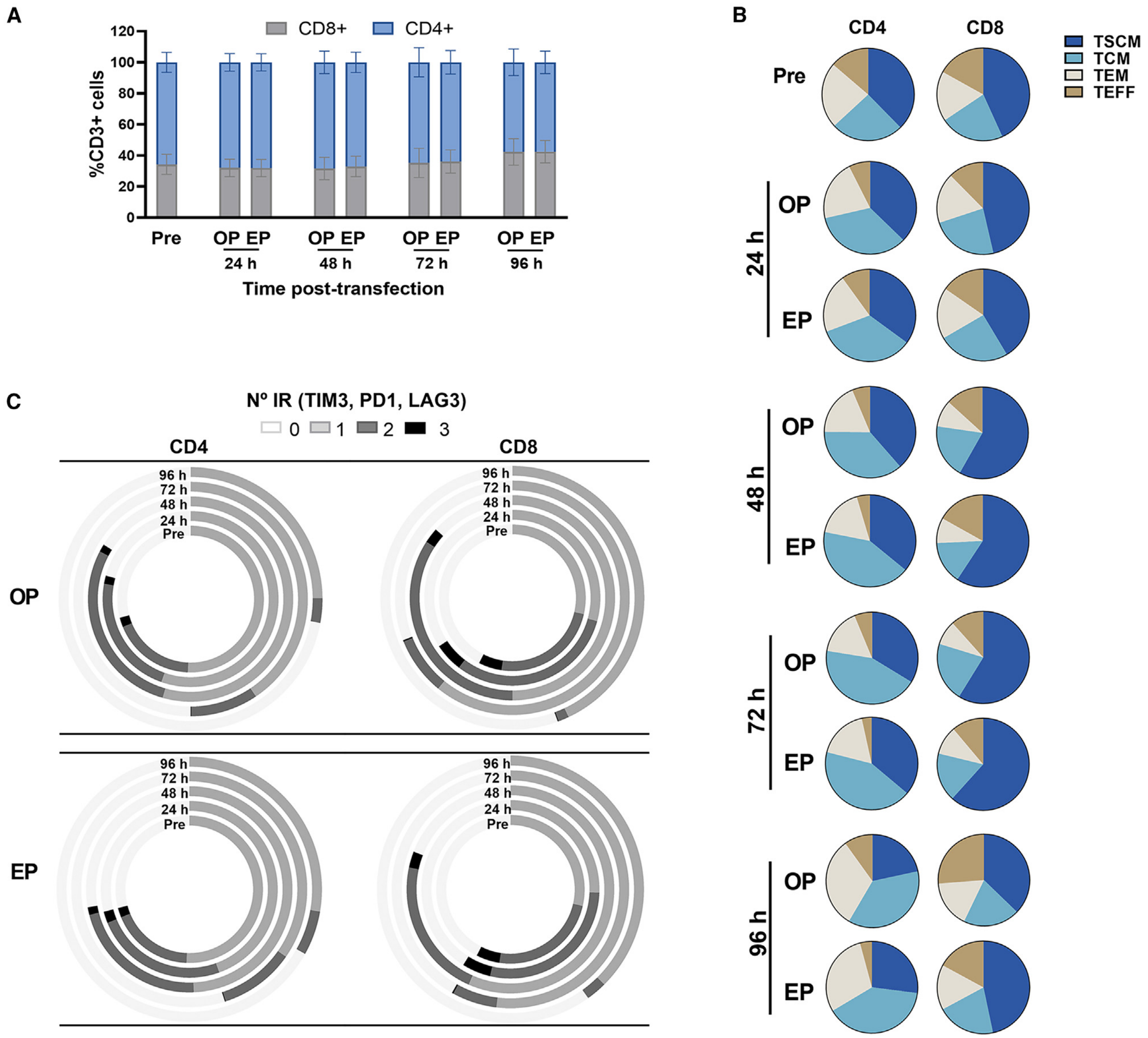

Figure 6. CAR-T cells generated using optoporation preserve a phenotype similar to electroporated CAR-T cells

(A) Proportion of $CD4^+$ and $CD8^+$ T cells in the $CD3^+$ T cell population at baseline conditions (pre) and at different time points after optoporation (OP) or electroporation (EP) with αPTPRZ1-CAR-mRNA, $n = 3$ independent donors. (B) Relative proportions of memory/effector $CD4^+CD3^+$ and $CD8^+CD3^+$ subsets in T cells before and after engineering via optoporation (OP) or electroporation (EP) with αPTPRZ1-CAR-mRNA. Data are shown as the mean of three independent donors. (C) Spice graphs showing the percentage of expression of 0 (white), 1 (light gray), 2 (dark gray), and 3 (black) exhaustion markers/inhibitory receptors (IRs) (TIM3, PD1, and LAG3) in $CD4^+CD3^+$ and $CD8^+CD3^+$ T cell subsets before and after engineering via optoporation (OP) or electroporation (EP) with αPTPRZ1-CAR-mRNA. Data are shown as the mean of 3 independent donors. TSCM: $CD45RA^+CCR7^+CD62L^+CD95^+$; T central memory (TCM): $CD45RA^-CCR7^+$; TEM: $CD45RA^-CCR7^-$ and T effector (TEFF): $CD45RA^+CCR7^-$.

supplemented with 10 mM HEPES, 1 mM sodium pyruvate, 5% p/s, 5% AB serum (Sigma), 2 mM L-glutamine/L-alanine (Bioswisstec AG), and 100 IU/mL hIL-2 (Proleukin). THP-1 cells (ATCC TIB-202) were cultured in RPMI-1640 supplemented with 10 mM HEPES, 1 mM sodium pyruvate, 5% p/s, and 10% iFBS. All cell lines were grown in a humidified incubator (37°C, 5% CO_2). Cell proliferation and viability were monitored using an Automated Countess III device (Invitrogen). Cells were regularly tested for *Mycoplasma* contamination using the MycoAlert PLUS Mycoplasma Detection Kit (Lonza).

Human T cells were obtained from buffy coats or human blood from healthy donors (Interregionale Blutspende SRK AG). T cells were isolated from buffy coats using the EasySep Direct Human T cell Isolation Kit (StemCell Technologies) and from whole blood using the RosetteSep Human T cell Enrichment Cocktail (StemCell

Technologies), followed by density gradient separation using Ficoll Paque Plus (Cytiva Life Sciences). Isolated T cells were diluted to 1×10^6 cells/mL and stimulated with Dynabeads Human T-Activator CD3/CD28 (Thermo Fisher Scientific) or human CD3/CD28 T cell Activation Beads (BioLegend) at a 1:1 ratio. Cells were cultured in RPMI-1640 supplemented with 10 mM HEPES, 1 mM sodium pyruvate, 5% p/s, 10% iFBS, and 30 IU/mL hIL-2 (T cell medium).

mRNA production

EGFP-mRNA (980 nt, N1-Methylpseudouridine/m1Ψ, CleanCap) was purchased from GenScript and stored at −80°C. Anti-CD19-CAR-mRNA (N1-Me-Pseudo UTP), encoding for αCD19-CAR comprising a high-affinity scFv (FMC63), was purchased from CATUG and stored at −80°C. Proprietary PTPRZ1-targeting VHH-CAR and IL13Rα2-targeting VHH-CAR were cloned into a proprietary mRNA production plasmid under the control of a promoter for T7 RNA polymerase.[42] The resultant plasmid was linearized by digestion with the restriction enzyme SalI (New England Biolabs). mRNA was transcribed using MegaScript T7 RNA polymerase (Thermo Fisher Scientific) with co-transcriptional capping using the CleanCap trinucleotide cap 1 analog (TriLink Biotechnologies). Uridine residues were fully substituted with N1-methylpseudouridine (m1Ψ) by using N1-methylpsuedouridine-5′- triphosphate (m1ΨTP) (TriLink Biotechnologies) in the place of uridine-5′-triphosphate in the reaction mixture. Poly(A) tailing procedure was performed after transcription using a Poly(A) Tailing Kit (Thermo Fisher Scientific). Finally, the mRNA was cleaned with the MEGAclear Transcription Clean-Up Kit (Thermo Fisher Scientific). The resultant mRNA was analyzed by gel electrophoresis, concentration and purity were determined using a Nanodrop device, and mRNA was precipitated, dried, and stored at −80°C until use.

Optoporation platform characteristics

The optoporation platform prototype was custom-built by Helbling Technik Wil AG (Figures 1B and S1). The setup consisted of a moving xy stage that can accommodate a plate (48-, 24-, 12-, or 6-well plate) where the movement speed and plate location can be controlled. The system allowed variation of the laser focal point, power, repetition rate and pulse length, meanwhile the laser spot pattern is predefined as circularly cropped. The optical setup included a pulsed laser with a pulse duration of 5–10 ns and tuned at a wavelength of 532 nm (CNI laser, GMP), a beam expander (Optograma), an ND filter (Thorlabs), three mirrors (Thorlabs), and a lens (Edmund Optics). The optomechanical components included a xy stage (Thorlabs), a z stage (Thorlabs), a mount for the beam expander (Newport), solid aluminum optical breadboards (Thorlabs) and a translating lens mount (Thorlabs).

Optoporation of human T cells

Human T cells containing Dynabeads were washed, resuspended in Opti-MEM (reduced serum medium, Gibco) and seeded into 48-well plates (1×10^6 cells per well, Corning, plastic bottom). Next, mRNA at the desired concentration (0.05 μg/μL, 5 μg in total) was immediately added to the cells and cells were run through the optoporation device using the indicated parameters. For mock-optoporation (NO RNA) control, cells were seeded directly in Opti-MEM medium without cargo to check the impact of the optoporation procedure in absence of cargo. The NO RNA control was performed for every tested condition such that efficiency delivery could be corrected for a shift in background fluorescence. An additional control was included to study mRNA delivery efficiency in the absence of Dynabeads (NO BEADS). Finally, the untreated control (no optoporation, no RNA) consisted of cells without cargo and that did not undergo the optoporation. In all conditions, T cells were seeded in a final volume of 100 μL and a quick spin-down of the plate was performed for cells to sediment. After optoporation, T cells were incubated overnight at 37°C with 5% CO_2 in complete T cell medium. For cytotoxicity assays, T cells were cultured in T cell medium without cytokine supplementation.

Optoporation of NK-92 and THP-1 cells

Dynabeads were added to cells in culture 30 min before optoporation at a 1:1 ratio. The next steps were performed similarly as described before. Briefly, cells were washed, resuspended in 100 μL Opti-MEM containing EGFP-mRNA (0.05 μg/μL) and transferred to a 48-well plate. Cells were quickly spin-down and plate was placed into the optoporation platform.

mRNA transfection by electroporation

After 48 h activation with Dynabeads at a 1:1 ratio as described above, Dynabeads were magnetically removed and activated T cells were washed with MaxCyte Electroporation buffer (Cytiva Life Sciences). We electroporated 2 × cells T cells with 5 μg of mRNA using ExPERT GTx (MaxCyte). Following electroporation, T cells were incubated overnight at 37°C with 5% CO_2 in complete T cell medium. For cytotoxicity assays, T cells were cultured in T cell medium without cytokine supplementation.

Flow cytometry

CAR surface expression was measured using biotinylated alpaca anti-human IgG F(ab')₂ (1:200, Jackson Immunoresearch) followed by PE-conjugated streptavidin (1:200, BD Biosciences). To determine αCD19-CAR expression, the Recombinant Human CD19 Fc Chimera Alexa Fluor 647 Protein (R&D Systems) was used. The LIVE/DEAD Fixable Near-IR Dead Cell Stain Kit or LIVE/DEAD Fixable Yellow Dead Cell Stain Kit (Invitrogen) were used to discriminate between live and dead cells. For the immunophenotyping of primary T cells, the following anti-human monoclonal antibodies were used: CD3-BV711 (1:250, BioLegend), CD8-AF700 (1:250, BioLegend), CD62L-PerCP-Cy5.5 (1:250, BioLegend), CD45RA-BV510 (1:250, BioLegend), CD95-AF647 (1:140, Bio Legend), CD25-BV605 (1:250, BioLegend), CD69-BV650 (1:140, BioLegend), CCR7-PE-Cy7 (1:140, BioLegend), PD1-BV421 (1:100, BioLegend), LAG3-BUV395 (1:100, BD), and TIM3-PE-Dazzle594 (1:100, BioLegend). Isotype controls were used for anti-PD1 (BioLegend), anti-LAG3 (BioLegend), and anti-TIM3 (BD Biosciences). The effector/memory T cell subsets were defined as follows: stem cell memory (TSCM): $CD45RA^+CCR7^+CD62L^+CD95^+$, central memory (TCM): $CD45RA^-CCR7^+$, effector memory (TEM): $CD45RA^-CCR7^-$, effector (TEFF): $CD45RA^+CCR7^-$. Naive cells

($CD45RA^{+}CCR7^{+}CD62L^{+}CD95^{-}$) were not found in any of the conditions.

For cytokine and granzyme production profiling, TNF-α-APC (1:100, BioLegend), IFN-γ-PE (1:100, BioLegend), IL-2-BV650 (1:100, BioLegend), and granzyme B-PE-TexasRed (1:100, BD) antibodies were used. Previously, T cells were activated for 4 h with CD3/CD28 Dynabeads in presence of Brefeldin A Solution (Thermo Fisher Scientific). Intracellular stainings were performed using the eBioscience Intracellular Fixation & Permeabilization Buffer Set (Invitrogen), following the manufacturer's recommendations.

To evaluate cytokine secretion in the supernatants, we used the LEGENDplex Human CD8/NK Panel (13-plex; BioLegend) following the manufacturer's instructions, and the data were analyzed using the LEGENDplex Data Analysis Software Suite (Qognit).

Cell staining was performed on ice and in the dark and cells were centrifuged at $400\times g$ for 5 min. Cells were washed with PBS + 3% FBS + 2 mM EDTA (wash buffer). Samples were acquired on a CytoFLEX LX cytometer (Beckman Coulter), and the data were analyzed using the FlowJo program (TreeStar) in the appropriate window after removal of doublets and dead cells. Samples were excluded from flow cytometry analyses when fewer than 100 events were recorded in the cell population of interest.

Flow cytometry-based killing assay

Antigen-positive (Ge518-PTPRZ1) and antigen-negative (Ge518-WT) tumor cells were stained using the CellTrace Far Red Cell Proliferation Kit (Invitrogen) and 2.5×10^{4} cells per well were seeded in a 96-well flat-bottom plate. The tumor cells were incubated for 2 h at 37°C with 5% CO_2 to allow them to adhere to the plate. Anti-PTPRZ1 RNA CAR-T cells generated by optoporation (CAR OP) or electroporation (CAR EP), and control mock-optoporated (mock OP)/mock-electroporated (mock EP) T cells were added to target cells at a 1:1 effector-to-target (E:T) ratio in T cell medium without cytokines. After 72 h of co-culture, cells were collected, and tumor cell death was measured by flow cytometry using a LIVE/DEAD Fixable Yellow Dead Cell Stain Kit (Invitrogen). Dead tumor cells were identified as Far Red^{+} LIVE/DEAD^{+} cells. The following formula was used to calculate the percentage of specific lysis by flow cytometry:

$$\%Specific\ lysis = \frac{\%Sample\ lysis - \%Baseline\ lysis}{100 - \%Baseline\ lysis} \times 100 \qquad \text{(Equation 1)}$$

Where *% Sample lysis* corresponds with lysis in the presence of mock or CAR-T cells and *% Baseline lysis* corresponds with lysis in the absence of T cells (tumor cells alone).

Incucyte-based killing assay

To determine the cytotoxicity of αCD19-CAR and αIL13Rα2-CAR T cells we used an Incucyte S3 Live-Cell Analysis System (Sartorius).Target tumor cells (Nalm-6 in the case of αCD19-CAR and Ge518-WT for αIL13Rα2-CAR T cells) and non-target tumor cells (Jurkat in the case of αCD19-CAR and Ge738 for αIL13Rα2-CAR T cells) expressing EGFP were seeded in a 96-well flat-bottom plate at a concentration of 5×10^{3} cells/well. Tumor cells were incubated for 2 h at 37°C with 5% CO_2 to allow adherence to the plate surface. Effector CAR-T cells generated by optoporation (CAR OP) or electroporation (CAR EP), and control mock-optoporated (mock OP)/mock-electroporated (mock EP) T cells were added to the tumor cells at a 1:4 E:T ratio, in the case of αCD19-CAR T cells, and 5:1 E:T ratio, in the case of αIL13Rα2-CAR T cells, in T cell medium without cytokines. Tumor cell growth was monitored for up to 40 h by real-time imaging, measuring the total fluorophore area per well every 2 h using the Incucyte mentioned. The data from each well were normalized to the first measurement (0 h).The percentage of specific lysis using Incucyte data was calculated according to the formula:

$$\%Specific\ lysis = \frac{0 - 100}{GFP\ area\ at\ \frac{40h}{0h}\ in\ presence\ of\ mock\ T\ cells} \times GFP\ area\ at\ \frac{40h}{0h}\ in\ presence\ of\ CAR - T\ cells + 100 \qquad \text{(Equation 2)}$$

Statistical analysis

Statistical analyses were performed using GraphPad Prism 9 (Dotmatics). Data were expressed as mean ± SEM. Each n represents an independent donor. The statistical test used is indicated in the corresponding Figure legend. Two-tailed Student's t tests to compare two groups with equal sample size, two-tailed Welch's t test to compare two groups with unequal sample size, and ANOVA tests for multiple comparisons were used.

DATA AVAILABILITY

All the data generated or analyzed in this study are included in this published article/supplemental information files. Raw data are available upon request from the corresponding author.

ACKNOWLEDGMENTS

We thank the flow cytometry core facility at Agora. Some diagrams were created using Biorender.

Funding: D.M. and N.M.P. were supported by the Swiss Innovation Agency Innosuisse through the grant 102.464 IP-LS. D.M. was supported by the ISREC Foundation.

AUTHOR CONTRIBUTIONS

N.M.P., L.H., Y.P., and D.M. conceived the study. N.M.P. designed and performed experiments and analyzed data. M.A.D. designed experiments and analyzed data. D.D., D.M.B., L.C., and C.B. contributed to experimental design. N.M.P., L.H., V.D., and D.M. wrote and edited the manuscript. D.M. supervised the study. All authors read and approved the final manuscript.

DECLARATION OF INTERESTS

D.M. is an inventor of patents related to CAR-T cell therapy, filed by the University of Pennsylvania, the Istituto Oncologico della Svizzera Italiana (IOSI), and the University

of Geneva. D.M. is a consultant for Limula SA and MPC Therapeutics SA. D.M. is the scientific co-founder and has an equity interest in Cellula Therapeutics SA. L.H. and Y.P. are co-founders and have an equity interest in Limula SA. C.B. and M.A.D. are employees of Limula.

SUPPLEMENTAL INFORMATION

Supplemental information can be found online at https://doi.org/10.1016/j.omtm.2025.101428.

REFERENCES

1. Elsallab, M., Ellithi, M., Lunning, M.A., D'Angelo, C., Ma, J., Perales, M.-A., Frigault, M., and Maus, M.V. (2024). Second primary malignancies after commercial CAR T-cell therapy: analysis of the FDA Adverse Events Reporting System. Blood *143*, 2099–2105. https://doi.org/10.1182/blood.2024024166.

2. Moretti, A., Ponzo, M., Nicolette, C.A., Tcherepanova, I.Y., Biondi, A., and Magnani, C.F. (2022). The Past, Present, and Future of Non-Viral CAR T Cells. Front. Immunol. *13*, 867013. https://doi.org/10.3389/fimmu.2022.867013.

3. Irving, M., Lanitis, E., Migliorini, D., Ivics, Z., and Guedan, S. (2021). Choosing the Right Tool for Genetic Engineering: Clinical Lessons from Chimeric Antigen Receptor-T Cells. Hum. Gene Ther. *32*, 1044–1058. https://doi.org/10.1089/hum.2021.173.

4. Levine, B.L., Pasquini, M.C., Connolly, J.E., Porter, D.L., Gustafson, M.P., Boelens, J.J., Horwitz, E.M., Grupp, S.A., Maus, M.V., Locke, F.L., et al. (2024). Unanswered questions following reports of secondary malignancies after CAR-T cell therapy. Nat. Med. *30*, 338–341. https://doi.org/10.1038/s41591-023-02767-w.

5. Dulery, R., Guiraud, V., Choquet, S., Thieblemont, C., Bachy, E., Barete, S., Todesco, È., Arnulf, B., Boissel, N., Baruchel, A., et al. (2025). T cell malignancies after CAR T cell therapy in the DESCAR-T registry. Nat. Med. 1–4. https://doi.org/10.1038/s41591-024-03458-w.

6. Ghilardi, G., Fraietta, J.A., Gerson, J.N., Van Deerlin, V.M., Morrissette, J.J.D., Caponetti, G.C., Paruzzo, L., Harris, J.C., Chong, E.A., Susanibar Adaniya, S.P., et al. (2024). T cell lymphoma and secondary primary malignancy risk after commercial CAR T cell therapy. Nat. Med. *30*, 984–989. https://doi.org/10.1038/s41591-024-02826-w.

7. Schutsky, K., Song, D.G., Lynn, R., Smith, J.B., Poussin, M., Figini, M., Zhao, Y., and Powell, D.J., Jr. (2015). Rigorous optimization and validation of potent RNA CAR T cell therapy for the treatment of common epithelial cancers expressing folate receptor. Oncotarget *6*, 28911–28928. https://doi.org/10.18632/oncotarget.5029.

8. Shah, P.D., Huang, A.C., Xu, X., Orlowski, R., Amaravadi, R.K., Schuchter, L.M., Zhang, P., Tchou, J., Matlawski, T., Cervini, A., et al. (2023). Phase I Trial of Autologous RNA-electroporated cMET-directed CAR T Cells Administered Intravenously in Patients with Melanoma and Breast Carcinoma. Cancer Res. Commun. *3*, 821–829. https://doi.org/10.1158/2767-9764.Crc-22-0486.

9. Foster, J.B., Barrett, D.M., and Karikó, K. (2019). The Emerging Role of In Vitro-Transcribed mRNA in Adoptive T Cell Immunotherapy. Mol. Ther. *27*, 747–756. https://doi.org/10.1016/j.ymthe.2019.01.018.

10. Chehelgerdi, M., and Chehelgerdi, M. (2023). The use of RNA-based treatments in the field of cancer immunotherapy. Mol. Cancer *22*, 106. https://doi.org/10.1186/s12943-023-01807-w.

11. Meister, H., Look, T., Roth, P., Pascolo, S., Sahin, U., Lee, S., Hale, B.D., Snijder, B., Regli, L., Ravi, V.M., et al. (2022). Multifunctional mRNA-Based CAR T Cells Display Promising Antitumor Activity Against Glioblastoma. Clin. Cancer Res. *28*, 4747–4756. https://doi.org/10.1158/1078-0432.CCR-21-4384.

12. Zhao, Y., Moon, E., Carpenito, C., Paulos, C.M., Liu, X., Brennan, A.L., Chew, A., Carroll, R.G., Scholler, J., Levine, B.L., et al. (2010). Multiple injections of electroporated autologous T cells expressing a chimeric antigen receptor mediate regression of human disseminated tumor. Cancer Res. *70*, 9053–9061. https://doi.org/10.1158/0008-5472.CAN-10-2880.

13. Zhao, Y., Zheng, Z., Cohen, C.J., Gattinoni, L., Palmer, D.C., Restifo, N.P., Rosenberg, S.A., and Morgan, R.A. (2006). High-efficiency transfection of primary human and mouse T lymphocytes using RNA electroporation. Mol. Ther. *13*, 151–159. https://doi.org/10.1016/j.ymthe.2005.07.688.

14. DiTommaso, T., Cole, J.M., Cassereau, L., Buggé, J.A., Hanson, J.L.S., Bridgen, D.T., Stokes, B.D., Loughhead, S.M., Beutel, B.A., Gilbert, J.B., et al. (2018). Cell engineering with microfluidic squeezing preserves functionality of primary immune cells *in vivo*. Proc. Natl. Acad. Sci. USA *115*, E10907–E10914. https://doi.org/10.1073/pnas.1809671115.

15. Raes, L., Stremersch, S., Fraire, J.C., Brans, T., Goetgeluk, G., De Munter, S., Van Hoecke, L., Verbeke, R., Van Hoeck, J., Xiong, R., et al. (2020). Intracellular Delivery of mRNA in Adherent and Suspension Cells by Vapor Nanobubble Photoporation. Nano-Micro Lett. *12*, 185. https://doi.org/10.1007/s40820-020-00523-0.

16. Wayteck, L., Xiong, R., Braeckmans, K., De Smedt, S.C., and Raemdonck, K. (2017). Comparing photoporation and nucleofection for delivery of small interfering RNA to cytotoxic T cells. J. Contr. Release *267*, 154–162. https://doi.org/10.1016/j.jconrel.2017.08.002.

17. Raes, L., Pille, M., Harizaj, A., Goetgeluk, G., Van Hoeck, J., Stremersch, S., Fraire, J.C., Brans, T., de Jong, O.G., Maas-Bakker, R., et al. (2021). Cas9 RNP transfection by vapor nanobubble photoporation for cell engineering. Mol. Ther. Nucleic Acids *25*, 696–707. https://doi.org/10.1016/j.omtn.2021.08.014.

18. Xiong, R., Raemdonck, K., Peynshaert, K., Lentacker, I., De Cock, I., Demeester, J., De Smedt, S.C., Skirtach, A.G., and Braeckmans, K. (2014). Comparison of gold nanoparticle mediated photoporation: vapor nanobubbles outperform direct heating for delivering macromolecules in live cells. ACS Nano *8*, 6288–6296. https://doi.org/10.1021/nn5017742.

19. Masters, B. (2008). Laser Manipulation of Cells and Tissues (Volume 82, Methods in Cell Biology). J. Biomed. Opt. *13*, 059902.

20. Krasieva, T., Chapman, C., LaMorte, V., Venugopalan, V., Berns, M., and Tromberg, B. (1998). Cell permeabilization and molecular transport by laser microirradiation. In Optical Investigations of Cells In Vitro and In Vivo (SPIE), pp. 38–44.

21. Paterson, L., Agate, B., Comrie, M., Ferguson, R., Lake, T., Morris, J., Carruthers, A., Brown, C.T., Sibbett, W., Bryant, P., et al. (2005). Photoporation and cell transfection using a violet diode laser. Opt. Express *13*, 595–600. https://doi.org/10.1364/OPEX.13.000595.

22. Terakawa, M., Ogura, M., Sato, S., Wakisaka, H., Ashida, H., Uenoyama, M., Masaki, Y., and Obara, M. (2004). Gene transfer into mammalian cells by use of a nanosecond pulsed laser-induced stress wave. Opt. Lett. *29*, 1227–1229. https://doi.org/10.1364/Ol.29.001227.

23. Yao, C.P., Zhang, Z.X., Rahmanzadeh, R., and Huettmann, G. (2008). Laser-based gene transfection and gene therapy. IEEE Trans. Nanobiosci. *7*, 111–119. https://doi.org/10.1109/Tnb.2008.2000742.

24. Tsukakoshi, M., Kurata, S., Nomiya, Y., Ikawa, Y., and Kasuya, T. (1984). A Novel Method of DNA Transfection by Laser Microbeam Cell Surgery. Appl. Phys. B *35*, 135–140. https://doi.org/10.1007/Bf00697702.

25. Raes, L., Van Hecke, C., Michiels, J., Stremersch, S., Fraire, J.C., Brans, T., Xiong, R., De Smedt, S., Vandekerckhove, L., Raemdonck, K., and Braeckmans, K. (2019). Gold Nanoparticle-Mediated Photoporation Enables Delivery of Macromolecules over a Wide Range of Molecular Weights in Human CD4+T Cells. Crystals *9*, 411. https://doi.org/10.3390/cryst9080411.

26. Urban, A.S., Fedoruk, M., Horton, M.R., Rädler, J.O., Stefani, F.D., and Feldmann, J. (2009). Controlled Nanometric Phase Transitions of Phospholipid Membranes by Plasmonic Heating of Single Gold Nanoparticles. Nano Lett. *9*, 2903–2908. https://doi.org/10.1021/nl901201h.

27. Siems, A., Weber, S.A.L., Boneberg, J., and Plech, A. (2011). Thermodynamics of nanosecond nanobubble formation at laser-excited metal nanoparticles. New J. Phys. *13*, 043018. https://doi.org/10.1088/1367-2630/13/4/043018.

28. Xiong, R., Samal, S.K., Demeester, J., Skirtach, A.G., De Smedt, S.C., and Braeckmans, K. (2016). Laser-assisted photoporation: fundamentals, technological advances and applications. Adv. Phys. X *1*, 596–620. https://doi.org/10.1080/23746149.2016.1228476.

29. Lapotko, D. (2009). Optical excitation and detection of vapor bubbles around plasmonic nanoparticles. Opt. Express *17*, 2538–2556. https://doi.org/10.1364/OE.17.002538.

30. Stewart, M.P., Langer, R., and Jensen, K.F. (2018). Intracellular Delivery by Membrane Disruption: Mechanisms, Strategies, and Concepts. Chem. Rev. *118*, 7409–7531. https://doi.org/10.1021/acs.chemrev.7b00678.

31. Maheshwari, S., van der Hoef, M., Prosperetti, A., and Lohse, D. (2018). Dynamics of Formation of a Vapor Nanobubble Around a Heated Nanoparticle. J. Phys. Chem. C Nanomater. Interfaces *122*, 20571–20580. https://doi.org/10.1021/acs.jpcc.8b04017.

32. Vermeulen, L.M.P., Fraire, J.C., Raes, L., De Meester, E., De Keulenaer, S., Van Nieuwerburgh, F., De Smedt, S., Remaut, K., and Braeckmans, K. (2018). Photothermally Triggered Endosomal Escape and Its Influence on Transfection Efficiency of Gold-Functionalized JetPEI/pDNA Nanoparticles. Int. J. Mol. Sci. *19*, 2400. https://doi.org/10.3390/ijms19082400.

33. Fraire, J.C., Houthaeve, G., Liu, J., Raes, L., Vermeulen, L., Stremersch, S., Brans, T., García-Díaz Barriga, G., De Keulenaer, S., Van Nieuwerburgh, F., et al. (2020). Vapor nanobubble is the more reliable photothermal mechanism for inducing endosomal escape of siRNA without disturbing cell homeostasis. J. Contr. Release *319*, 262–275. https://doi.org/10.1016/j.jconrel.2019.12.050.

34. Foulkes, R., Man, E., Thind, J., Yeung, S., Joy, A., and Hoskins, C. (2020). The regulation of nanomaterials and nanomedicines for clinical application: current and future perspectives. Biomater. Sci. *8*, 4653–4664. https://doi.org/10.1039/d0bm00558d.

35. Ziefuss, A.R., Reich, S., Reichenberger, S., Levantino, M., and Plech, A. (2020). structural kinetics of picosecond laser-induced heating and fragmentation of colloidal gold spheres. Phys. Chem. Chem. Phys. *22*, 4993–5001. https://doi.org/10.1039/c9cp05202j.

36. Davies, K. (2024). On the Charge: MaxCyte Celebrates Role in Sickle Cell Therapy Breakthrough. https://www.genengnews.com/topics/genome-editing/on-the-charge-maxcyte-celebrates-role-in-sickle-cell-therapy-breakthrough/.

37. Arcangeli, S., Bove, C., Mezzanotte, C., Camisa, B., Falcone, L., Manfredi, F., Bezzecchi, E., El Khoury, R., Norata, R., Sanvito, F., et al. (2022). CAR T cell manufacturing from naive/stem memory T lymphocytes enhances antitumor responses while curtailing cytokine release syndrome. J. Clin. Investig. *132*, e150807. https://doi.org/10.1172/JCI150807.

38. Arcangeli, S., Falcone, L., Camisa, B., De Girardi, F., Biondi, M., Giglio, F., Ciceri, F., Bonini, C., Bondanza, A., and Casucci, M. (2020). Next-Generation Manufacturing Protocols Enriching T(SCM) CAR T Cells Can Overcome Disease-Specific T Cell Defects in Cancer Patients. Front. Immunol. *11*, 1217. https://doi.org/10.3389/fimmu.2020.01217.

39. Biasco, L., Izotova, N., Rivat, C., Ghorashian, S., Richardson, R., Guvenel, A., Hough, R., Wynn, R., Popova, B., Lopes, A., et al. (2021). Clonal expansion of T memory stem cells determines early anti-leukemic responses and long-term CAR T cell persistence in patients. Nat. Cancer *2*, 629–642. https://doi.org/10.1038/s43018-021-00207-7.

40. Blaeschke, F., Stenger, D., Kaeuferle, T., Willier, S., Lotfi, R., Kaiser, A.D., Assenmacher, M., Döring, M., Feucht, J., and Feuchtinger, T. (2018). Induction of a central memory and stem cell memory phenotype in functionally active CD4(+) and CD8(+) CAR T cells produced in an automated good manufacturing practice system for the treatment of CD19(+) acute lymphoblastic leukemia. Cancer Immunol. Immunother. *67*, 1053–1066. https://doi.org/10.1007/s00262-018-2155-7.

41. Mehta, P.H., Fiorenza, S., Koldej, R.M., Jaworowski, A., Ritchie, D.S., and Quinn, K.M. (2021). T Cell Fitness and Autologous CAR T Cell Therapy in Haematologic Malignancy. Front. Immunol. *12*, 780442. https://doi.org/10.3389/fimmu.2021.780442.

42. Martinez Bedoya, D., Marinari, E., Davanture, S., Castillo Cantero, L., Erraiss, S., Dockerill, M., Barluenga, S., Winssinger, N., Schaller, K., Bijlenga, P., et al. (2024). PTPRZ1-Targeting RNA CAR T Cells Exert Antigen-Specific and Bystander Antitumor Activity in Glioblastoma. Cancer Immunol. Res. *12*, 1718–1735. https://doi.org/10.1158/2326-6066.Cir-23-1094.

43. Dutoit, V., Herold-Mende, C., Hilf, N., Schoor, O., Beckhove, P., Bucher, J., Dorsch, K., Flohr, S., Fritsche, J., Lewandrowski, P., et al. (2012). Exploiting the glioblastoma peptidome to discover novel tumour-associated antigens for immunotherapy. Brain *135*, 1042–1054. https://doi.org/10.1093/brain/aws042.

44. Migliorini, D., Dutoit, V., Allard, M., Grandjean Hallez, N., Marinari, E., Widmer, V., Philippin, G., Corlazzoli, F., Gustave, R., Kreutzfeldt, M., et al. (2019). Phase I/II trial testing safety and immunogenicity of the multipeptide IMA950/poly-ICLC vaccine in newly diagnosed adult malignant astrocytoma patients. Neuro Oncol. *21*, 923–933. https://doi.org/10.1093/neuonc/noz040.

45. Harizaj, A., Wels, M., Raes, L., Stremersch, S., Goetgeluk, G., Brans, T., Vandekerckhove, B., Sauvage, F., De Smedt, S.C., Lentacker, I., and Braeckmans, K. (2021). Photoporation with Biodegradable Polydopamine Nanosensitizers Enables Safe and Efficient Delivery of mRNA in Human T Cells. Adv. Funct. Mater. *31*, 2102472. https://doi.org/10.1002/adfm.202102472.

46. Zhang, M., Ma, Z., Selliah, N., Weiss, G., Genin, A., Finkel, T.H., and Cron, R.Q. (2014). The impact of Nucleofection® on the activation state of primary human CD4 T cells. J. Immunol. Methods *408*, 123–131. https://doi.org/10.1016/j.jim.2014.05.014.

47. Maus, M.V., Haas, A.R., Beatty, G.L., Albelda, S.M., Levine, B.L., Liu, X., Zhao, Y., Kalos, M., and June, C.H. (2013). T Cells Expressing Chimeric Antigen Receptors Can Cause Anaphylaxis in Humans. Cancer Immunol. Res. *1*, 26–31. https://doi.org/10.1158/2326-6066.Cir-13-0006.

48. Hanssens, H., Meeus, F., Gesquiere, E.L., Puttemans, J., De Vlaeminck, Y., De Veirman, K., Breckpot, K., and Devoogdt, N. (2024). Anti-Idiotypic VHHs and VHH-CAR-T Cells to Tackle Multiple Myeloma: Different Applications Call for Different Antigen-Binding Moieties. Int. J. Mol. Sci. *25*, 5634. https://doi.org/10.3390/ijms25115634.

49. Ackaert, C., Smiejkowska, N., Xavier, C., Sterckx, Y.G.J., Denies, S., Stijlemans, B., Elkrim, Y., Devoogdt, N., Caveliers, V., Lahoutte, T., et al. (2021). Immunogenicity Risk Profile of Nanobodies. Front. Immunol. *12*, 632687. https://doi.org/10.3389/fimmu.2021.632687.

OMTM, Volume *33*

Supplemental information

Magnetic bead-sensitized optoporation coupled with antibodies-based activation for mRNA CAR-T cell manufacturing

Noelia Maldonado-Pérez, Marie-Agnès Doucey, Dzhangar Dzhumashev, Darel Martínez Bedoya, Luis Castillo Cantero, Caroline Boudousquie, Yann Pierson, Luc Henry, Valérie Dutoit, and Denis Migliorini

Table S1. Summary of the optoporation parameters used after optimization.

Delivery parameter	**Range**	**Optimal condition**
Pulse energy	1 - 90 µJ	90 µJ
Repetition rate	100 - 1000 Hz	1000 Hz
Scanning speed	10 - 200 mm/s	80 mm/s
Longitudinal spot location	0 - 18 mm	13.9 mm
Diameter lens	1.2 - 9.6 mm	1.2 mm
Spot size	1.32 - 10.58 µm	10.58 µm

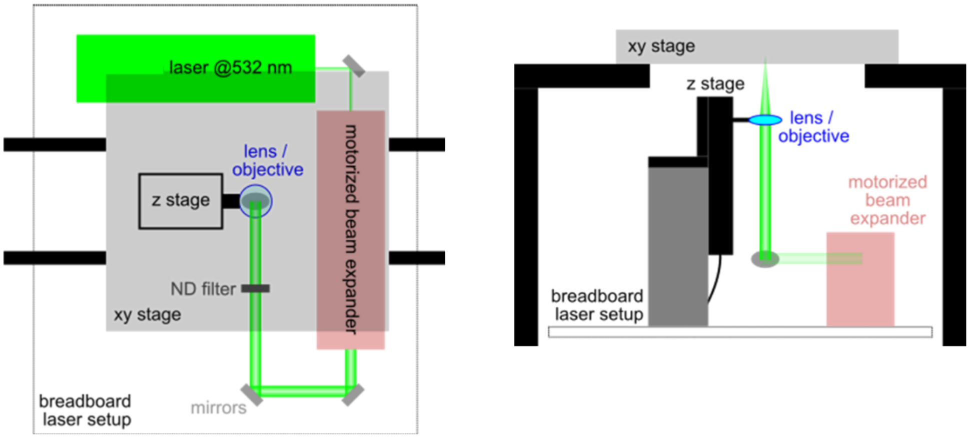

Figure S1. Optical scheme of the optoporation platform. The xy stage and laser setup are on separate breadboards. The laser setup counts with a beam expander, a lens, a ND filter to allow very low pulse energies (nJ range or below) and three mirrors.

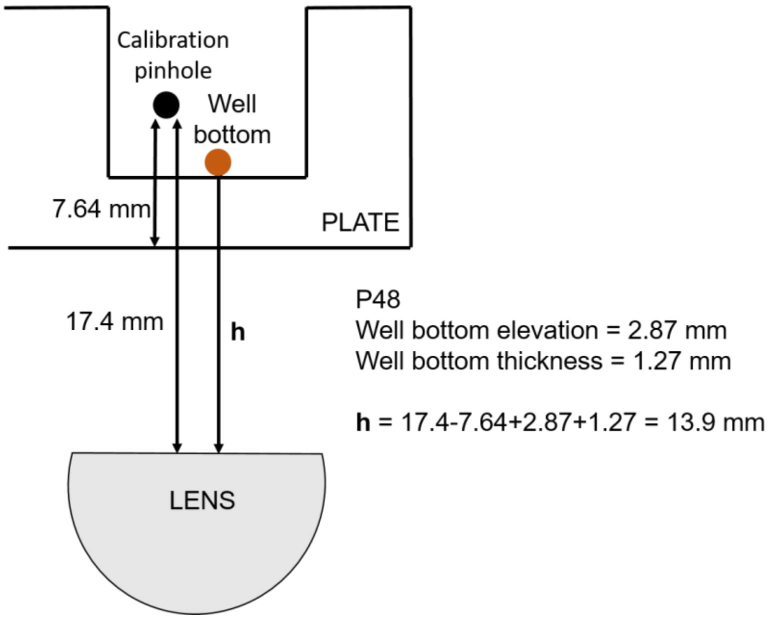

Figure S2. **Longitudinal spot location (LSL) estimation to locate the laser spot at the bottom of the well of a 48 well plate.** The input value for LSL is the result of the calculation of distance from lens to well bottom (h), which is based on the reference distances to the pinhole provided by the manufacturer.

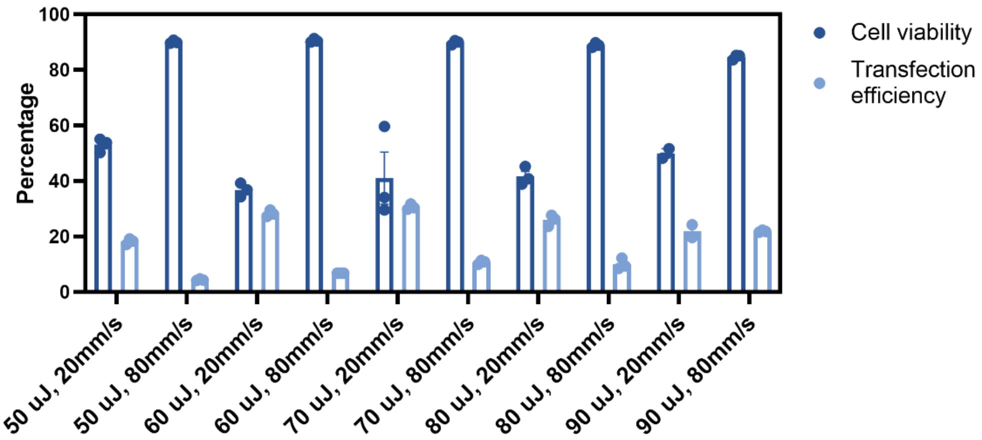

Figure S3. Optimization of scanning speed and energy pulse. Cell viability (dark blue) measured by flow cytometry 24 hours post-optoporation by staining of cells with a viability marker and efficiency of optoporation (light blue) measured by flow cytometry and reflected by the percentage of eGFP+ cells. Shown are mean cell viability and optoporation efficiency from 3 technical replicates ± SEM.

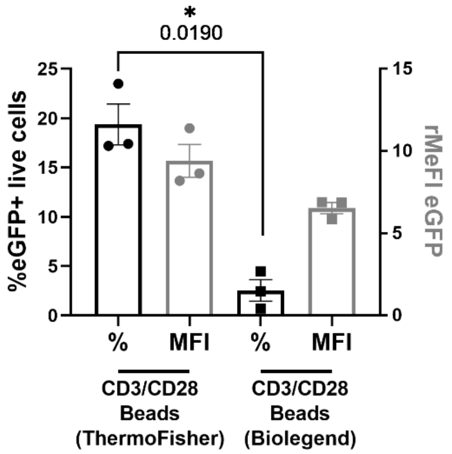

Figure S4. Magnetic bead-sensitized optoporation efficiency is dependent on the origin of CD3/CD28 magnetic beads. Primary human T cells were activated with human CD3/CD28 Dynabeads from ThermoFisher or human CD3/CD28 T Cell Activation Beads from Biolegend before optoporation of eGFP-mRNA. Transfection efficiency 24 hours post-optoporation is expressed as the percentage of eGFP+ live cells (left y-axis, black) and the relative median fluorescence intensity (rMeFI) relative to eGFP- cells (right y-axis, grey), n=3 independent donors. Data are shown as mean ± SEM.

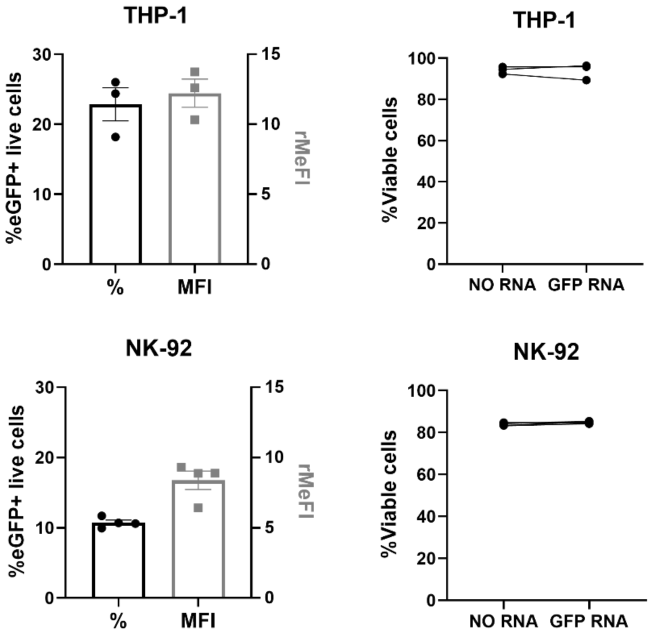

Figure S5. Monocyte and NK cell lineages are efficiently optoporated. THP-1 cells (top row) and NK cells (bottom row) optoporation efficiency and viability were measured by flow cytometry. Transfection efficiency expressed as percentage of eGFP+ cells among live cells (left y-axis, black) and relative median fluorescence intensity (rMeFI) relative to eGFP- cells (right y-axis, grey) is shown 24 hours post-optoporation. Cell viability was measured 24 hours post-optoporation by staining with a cell viability marker Data are shown as mean ± SEM.

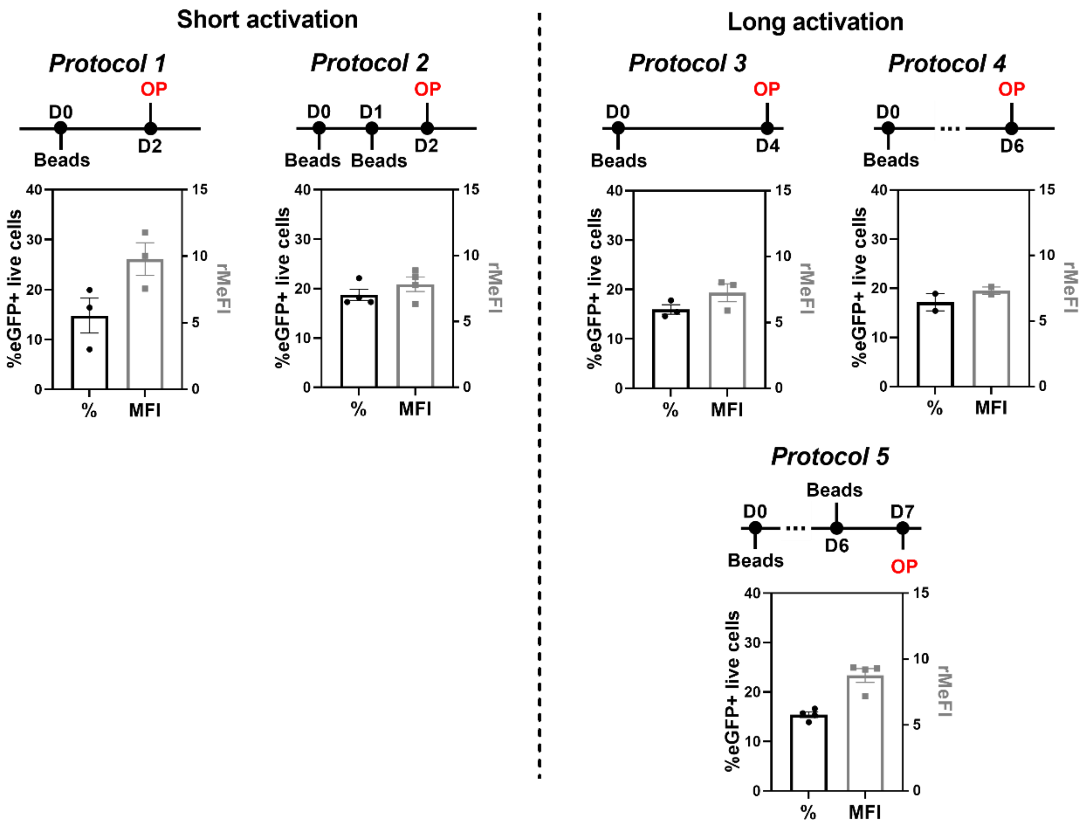

Figure S6. Impact of T cell activation time on mRNA optoporation efficacy. Primary human T cells were activated with CD3/CD28 Dynabeads (Beads) for different times before performing optoporation (OP) of eGFP-mRNA. Transfection efficiency 24 hours post-optoporation is expressed as the percentage of eGFP+ live cells (left y-axis, black) and the relative median fluorescence intensity (rMeFI) relative to eGFP- cells (right y-axis, grey) of different conditions. Data are shown as mean ± SEM.

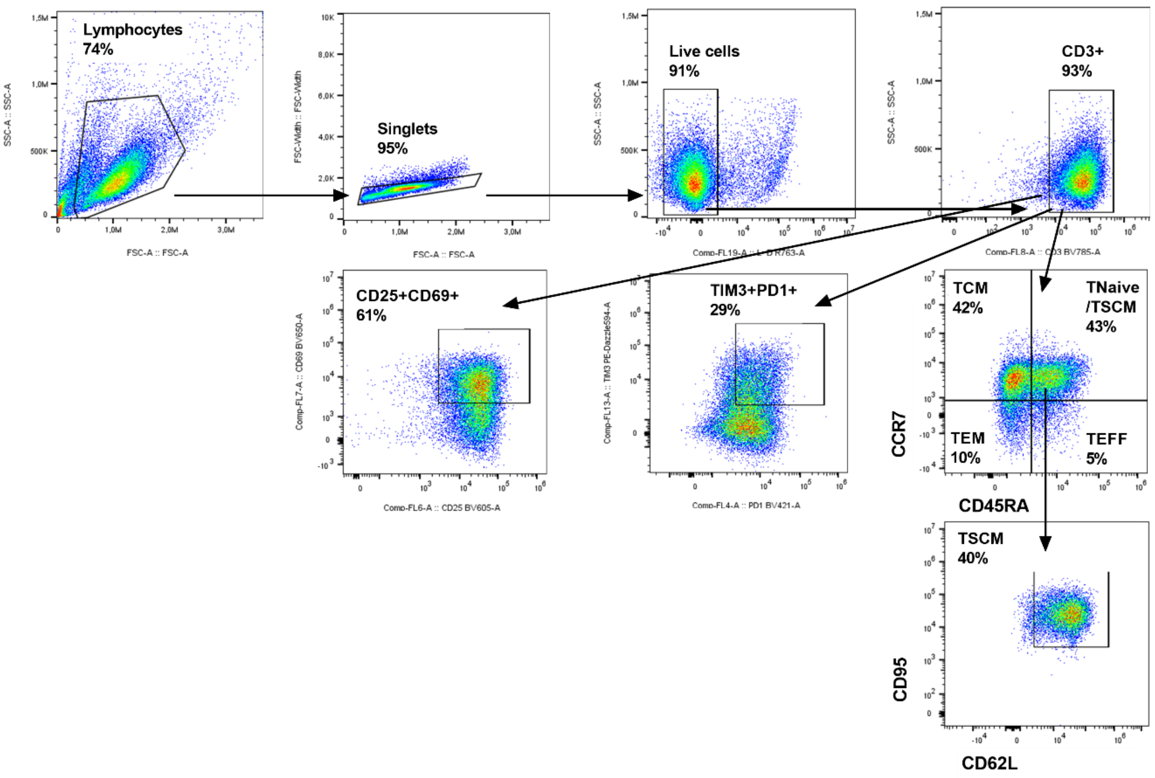

Figure S7. Gating strategy. Flow cytometry data analysis followed to obtain the different T cell populations.

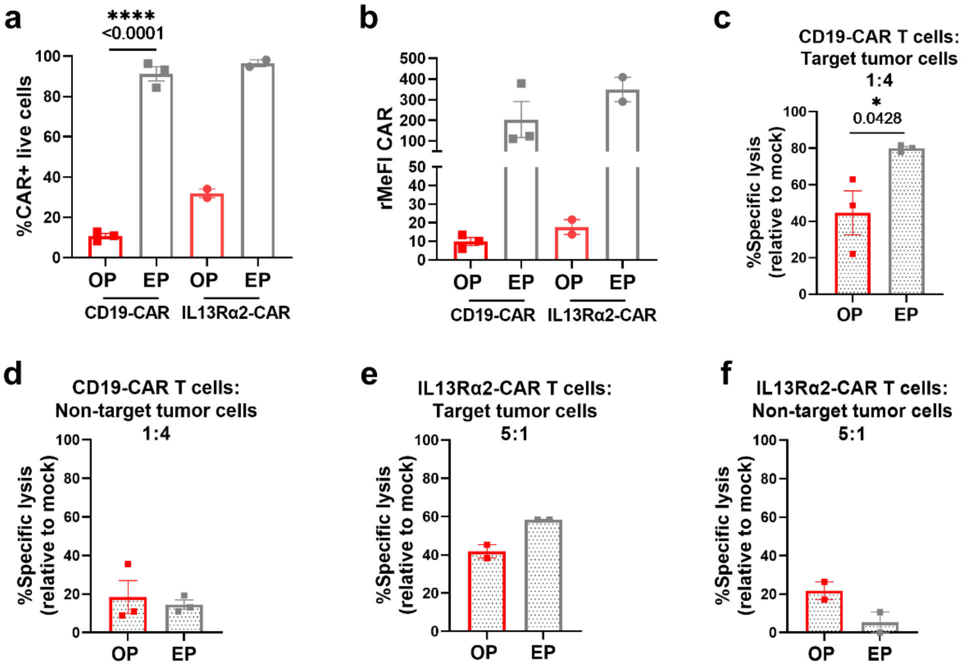

Figure S8. **Generation of different functional CAR T cells through mRNA optoporation. a)** Percentage of CAR+ T cells (left) and **b)** relative median fluorescence intensity (rMeFI) relative to CAR- cells (right) after optoporation (OP) and electroporation (EP) with αCD19-CAR-mRNA and αIL13Rα2-CAR-mRNA, n=2-3 independent donors. **c)** Percentage of specific lysis of αCD19-CAR-T cells generated by optoporation (OP) or electroporation (EP) (diluted to obtain the same percentage of CAR+ T cells) against target tumor cells (Nalm6) and **d)** non-target tumor cells (Jurkat) at E:T ratio 1:4 determined by an IncuCyte S3 system at 40 h of co-culture, n=3 independent donors. **e)** Percentage of specific lysis of αIL13Rα2-CAR-T cells generated by optoporation (OP) or electroporation (EP) (diluted to obtain the same percentage of CAR+ T cells) against target tumor cells (Ge518-WT) and **f)** non-target tumor cells (GE738) at E:T ratio 5:1 determined by an IncuCyte S3 system at 40 h of co-culture, n=2 independent donors. Data are shown as mean ± SEM. Statistics are based on paired, two-tailed Student´s t-test (a,c), *p<0.05, ****p<0.0001. The percentage of specific lysis was calculated according to the formula: (0-100)/(GFP area at 40h/0h in presence of mock T cells-0)*(GFP area at 40h/0h in presence of CAR-T cells)+100.

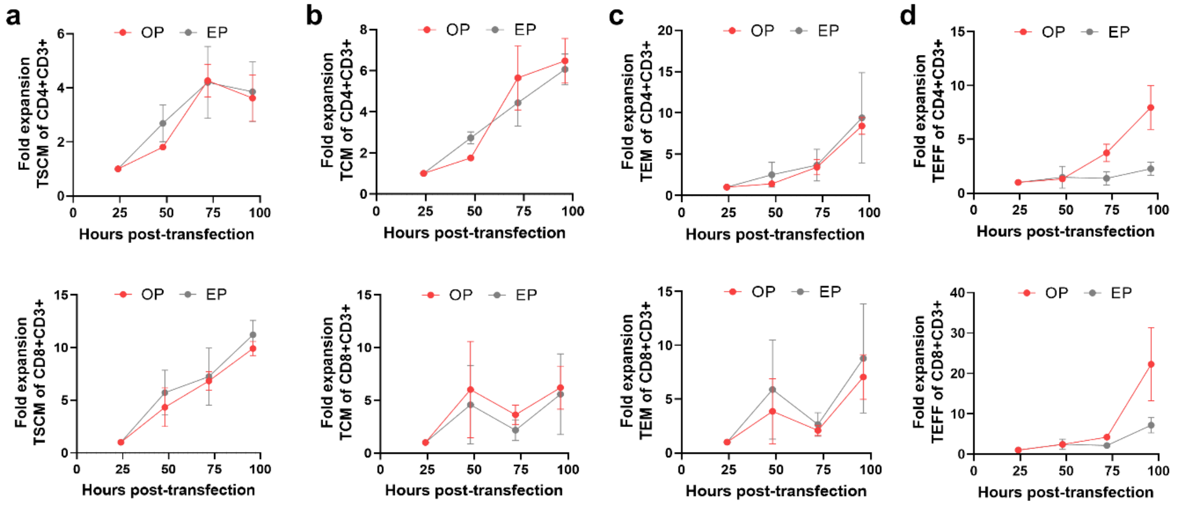

Figure S9. **Frequencies of T cell subpopulations in CAR-T cells generated via optoporation are comparable to electroporation. a)** Fold expansion of stem cell memory T cells (TSCM) CD4+CD3+ (upper graph) and TSCM CD8+CD3+ (lower graph) over time after optoporation (OP) or electroporation (EP) of αPTPRZ1-CAR mRNA. **b)** Fold expansion of central memory T cells (TCM) CD4+CD3+ (upper graph) and TCM CD8+CD3+ (lower graph) over time after optoporation (OP) or electroporation (EP) of αPTPRZ1-CAR mRNA. **c)** Fold expansion of effector memory T cells (TEM) CD4+CD3+ (upper graph) and TEM CD8+CD3+ (lower graph) over time after optoporation (OP) or electroporation (EP) of αPTPRZ1-CAR mRNA. **d)** Fold expansion of effector T cells (TEFF) CD4+CD3+ (upper graph) and TEFF CD8+CD3+ (lower graph) over time after optoporation (OP) or electroporation (EP) of αPTPRZ1-CAR mRNA. Cell counts were determined by Trypan blue exclusion and normalized to the initial number of seeded cells at 24 hours post-transfection, n=3 independent donors. Data are shown as mean ± SEM.
